# Structural Basis for Potent Inhibition of Human DHODH by Quinoline-4-carboxylic Acid Derivatives

**DOI:** 10.1021/acsomega.5c12978

**Published:** 2026-04-27

**Authors:** Jéssika de Oliveira Viana, Tayná Rodrigues Olegário, Marília Cecília da Silva, Rodrigo Cristiano, Euzébio Guimarães Barbosa, Aline Dias da Purificação, Marina Sena Mendes, Diogo Boerin, Maria Cristina Nonato, Karen Cacilda Weber, Claudio Gabriel Lima-Junior

**Affiliations:** † Computational Quantum Chemistry Laboratory, Department of Chemistry, Federal University of Paraíba, João Pessoa 58051-900, Paraíba, Brazil; ‡ Medicinal Organic Synthesis Laboratory of Paraíba (LASOM-PB), Department of Chemistry, Federal University of Paraíba, João Pessoa 58051-900, Paraíba, Brazil; § Computational Pharmaceutical Chemistry Laboratory, Faculty of Pharmacy, Federal University of Rio Grande do Norte, Natal 59078-970, Rio Grande do Norte, Brazil; ∥ Center for the Research and Advancement of Fragments and Molecular Targets (CRAFT), School of Pharmaceutical Sciences of Ribeirão Preto, University of São Paulo, Ribeirão Preto 14040-903, São Paulo, Brazil

## Abstract

Quinoline-4-carboxylic acid derivatives are privileged scaffolds in drug discovery, with potential as inhibitors of human dihydroorotate dehydrogenase (*Hs*DHODH), a clinically validated target in cancer and autoimmune diseases. Here, we report computational and structural studies of a series of 6-fluoro-2-(aryl)­quinoline-4-carboxylic acids, focusing on their mechanistic basis of inhibition. Molecular docking and molecular dynamics simulations predicted stable binding of compounds **2d** and **2e** within the *Hs*DHODH binding site. These findings, corroborated by MM-PBSA free energy calculations, suggest a solvent-assisted anchoring mechanism that differs from the binding mode of brequinar. Enzymatic assays confirmed nanomolar potency for compound **2d** (IC_50_ = 27 ± 1 nM), comparable to brequinar, while other derivatives displayed micromolar activity. Crucially, X-ray crystallography of the **2d**–*Hs*DHODH complex provided direct structural confirmation of the computationally predicted binding mode. In parallel, ADME modeling predicted a lower Log *P* for **2d** compared with brequinar, pointing to more favorable physicochemical properties. Together, these results establish compound **2d** as a potent and drug-like *Hs*DHODH inhibitor and illustrate how integrated computational and structural approaches can elucidate binding determinants of known scaffolds and guide future inhibitor design.

## Graphical abstract

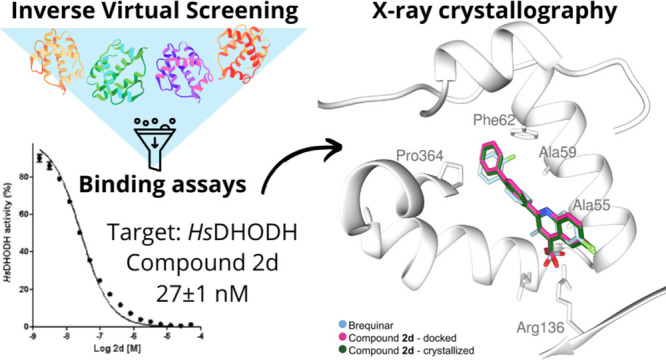

## 1 Introduction

Quinoline derivatives represent a chemically versatile class of compounds with broad pharmacological potential. Their fused aromatic scaffold provides a favorable framework for interactions with biological targets, contributing to their widespread application in medicinal chemistry. Notably, quinolines have been explored as antimicrobial, antiparasitic, anti-inflammatory, and anticancer agents, among other therapeutic uses.^1^ Their structural diversity can be easily expanded through well-established synthetic routes, allowing for the rational design and optimization of novel bioactive molecules. This combination of pharmacological relevance and synthetic accessibility makes quinoline derivatives particularly attractive for drug discovery efforts, encouraging further investigations into their mechanisms of action and therapeutic applications.^2^

Among quinoline derivatives, quinoline-4-carboxylic acid (4-QC) derivatives have displayed a wide spectrum of biologically relevant activities underpinned by distinct molecular mechanisms. Compounds bearing a methyl sulfonyl-phenyl moiety at C-2, such as analogs evaluated by Zarghi et al., are potent and selective cyclooxygenase-2 (COX-2) inhibitors, achieving IC_50_ values as low as 0.043 μM, more potent than the reference compound, celecoxib, and selectivity indexes exceeding 500.^3^ 2-Aryl-4-QC hydrazide–hydrazones have shown activity against both Gram-positive and Gram-negative bacteria, while nitro-substituted congeners exhibited strong antifungal and antibacterial effects, with MICs comparable to reference drugs, and low hemolytic toxicity.^4^ 7-Chloro-6-fluoro-2-phenyl-quinoline-4-carboxylic acid analogs displayed superior anticancer activity compared to doxorubicin in MCF-7, HeLa, Hep-2 and other carcinoma cell lines, inducing apoptotic DNA fragmentation and likely targeting human topoisomerase IIα.^5^

Importantly, fluorinated quinoline-4-carboxylates have also been identified as inhibitors of human dihydroorotate dehydrogenase (*Hs*DHODH),^6^ thereby disrupting de novo pyrimidine biosynthesis. Notably, the 6-fluoro-2-fluorobiphenyl-quinoline-4-carboxylic acid sodium salt, known as brequinar, acts through this mechanism and has been investigated for anticancer and antiviral applications.^7,8^ Biphenyl ether analogs such as C44 that was structurally characterized by X-ray crystallography and brequinar itself achieved nanomolar DHODH inhibition.^7^ Derivatives using protocatechuic aldehyde as precursor of 2-substituted quinoline-4-carboxylic acids containing two alkyl/aryl ether linkages were the most effective *Hs*DHODH inhibitors, while vanillin derivatives had high cytotoxic activity against MCF-7 and A375 cancer cells.^9^

Quinoline-4-carboxylic acid derivatives related to this scaffold have been previously reported as DHODH inhibitors, including early studies describing 2-arylquinoline analogues with antitumor activity.^10^ However, detailed structural information describing how simplified members of this class interact with the HsDHODH binding site remains limited. In particular, the molecular determinants governing their affinity and the role of solvent-mediated interactions in stabilizing ligand binding have not been extensively characterized.

In our ongoing efforts of structural simplification and low-cost synthesis, we synthesized a series of 6-fluoro-2-aryl-quinoline-4-carboxylic acids by combining 5-fluoroisatin and methyl aryl ketones with different substitution patterns, which has also shown antimicrobial and larvicidal potential.^11^

The identification of molecular targets represents a pivotal stage in the development of bioactive derivatives, as it enables a deeper understanding of their mechanisms of action and supports the rational optimization of therapeutic potential. Computational target prediction has emerged as a valuable approach to address this challenge, allowing researchers to predict potential protein targets for small molecules based on the chemical similarity principle.^12,13^ Among the available strategies, ligand-based inverse virtual screening (LB-IVS) allows for the rapid and cost-effective identification of candidate targets based on existing biochemical and structural data. This computational technique not only facilitates the drug repurposing of known compounds but also aids in uncovering novel target–compound interactions, which may reveal previously unrecognized therapeutic opportunities.^14^ Web servers and structure alignment and similarity scripts have been reported in the literature focusing on the identification of the mechanism of action and activity of query compounds based on ligand structure.^15^ By integrating LB-IVS with experimental validation, researchers can refine drug discovery pipelines, improving the selection of promising lead compounds and accelerating the development of new treatments across various diseases.

In this study, to gain mechanistic insights into molecular recognition of a series of 6-fluoro-2-(aryl)­quinoline-4-carboxylic acid derivatives (see Figure 1), we applied ligand-based inverse virtual screening (LB-IVS) which pointed human dihydroorotate dehydrogenase (*Hs*DHODH) as the top-ranked biological target. Notably, our query molecule in the LB-IVS, **2d**, was previously reported as a DHODH inhibitor,^10^ but without detailed structural insights. In this study, we validate and expand on previous findings by uncovering the molecular basis of potent inhibition through docking, molecular dynamics, and free energy calculations. Consistent with these insights, enzymatic assays demonstrated that compound **2d** inhibits *Hs*DHODH with an IC_50_ comparable to brequinar and substantially lower than teriflunomide, while the remaining compounds in the series displayed inhibition only at micromolar concentrations. To further confirm the predicted binding modes, we obtained X-ray crystal structures of the complex between two compounds and *Hs*DHODH, providing direct structural evidence for our findings. Additionally, ADME predictions suggested that our most potent compounds have lower Log *P* values than brequinar, indicating improved physicochemical properties. Together, these findings provide structural and mechanistic insight into the interaction of quinoline-4-carboxylic acids with *Hs*DHODH and highlight compound **2d** as a promising scaffold for further optimization of *Hs*DHODH inhibitors.

**Figure 1:**
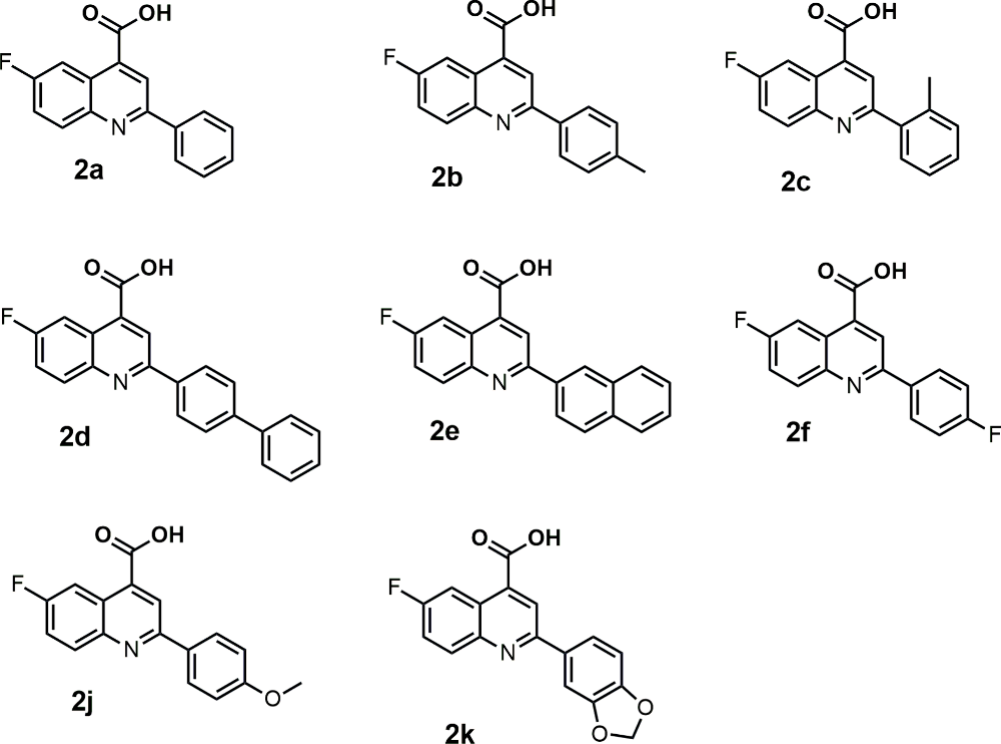
Chemical structures of the 6-fluoro-2-(aryl)­quinoline-4-carboxylic acid derivatives under study.

## 2 Materials and Methods

### 2.1 Geometry Optimization of Ligands

The two-dimensional structures of the 6-fluoro-2-(aryl)­quinoline-4-carboxylic acid series (**2a**–**f**, **2j** and **2k**) synthesized and characterized by Olegário et al.^11^ (see Figure 1), as well as the ones of reference DHODH ligands brequinar and teriflunomide, were modeled with MarvinSketch v.23.17 program (http://www.chemaxon.com). OpenBabel^16^ was employed to adjust protonation states for a pH of 7.4, while MOPAC software was used to optimize each molecular geometry employing the PM6 semiempirical method.^17^

### 2.2 Ligand-Based Inverse Virtual Screening (LBIVS)

#### 2.2.1 Target Fishing in Web Servers

Aiming to identify a putative target for the ligands under study, three target fishing servers were used, each one based on a different approach: SwissTargetPrediction, which is based on similarity with known ligands,^18^ Similarity ensemble approach (SEA) based on molecular fingerprints prediction models^19^ and TargetNet, also based on molecular fingerprints prediction models but with an integrated deep learning-based algorithm.^20^ Compound **2d** SMILES code was used as the input file for searching these web-based tools. In the SwissTargetPrediction Web server, the *Homo sapiens* organism was chosen as the starting point, and the identification of the preferred target for the compound was calculated in terms of probability. Through the TargetNet webtool, the models included to perform this search were based on an AUC ≥ 9.0 using ECFP4 fingerprint type, and the target identification was also obtained as a probability. From the SEA server, the results about the best target were given by the correlation of the maximum Tanimoto coefficient (MaxTC) with the P-value.

#### 2.2.2 Ad-Hoc Ligand-Based Inverse Virtual Screening

An ad-hoc ligand-based inverse virtual screening was performed using a comprehensive database of all crystallized ligands created by downloading their structures from the Ligand Expo repository (http://ligand-expo.rcsb.org/ld-download.html). The 3D structure of compound **2d** was then compared against this entire database using the LS-Align structural comparison tool.^21^ The PC-Score8Q metric was employed to quantify structural and chemical similarity, and the top-scoring ligands were selected. The target to which these top-scoring ligands bind was considered the probable target for compound **2d**, guiding further in silico and biological testing.

### 2.3 Molecular Docking

#### 2.3.1 Protocol Evaluation Using Redocking

Initially, redocking was performed using the three-dimensional structure of the identified target, *Hs*DHODH (PDB ID: 4JTU), complexed with the quinoline compound 6-bromo-2-{4-[(2*R*)-butan-2-yl]­phenyl}-3-methylquinoline-4-carboxylic acid (**JTU**). The protein structure underwent preparation by removing water molecules and assigning charges using the UCSF Chimera program.^22^ The Web server H++ was used for adding hydrogens in protein, considering a pH of 7.4 for charged residue protonation. The cofactor was retained at the binding site based on studies demonstrating its interaction with inhibitors.^23^ Redocking with flexible ligands was performed using Autodock Vina,^24^ employing Vina score, and full flexibility ligands for GOLD^25^ using four scoring functions: ASP, ChemPLP, Chemscore, and Goldscore. For Vina and GOLD, the grid was centered at 15 Å from the center of mass of the crystallized ligand. The best redocking poses obtained from Vina and GOLD were utilized as input data in the UCSF Chimera program,^22^ to calculate the root mean square deviation (RMSD) with the crystallized PDB ligand (**JTU**) serving as a fixed reference. A value ranging between 0.0 and 2.0 Å is indicative of a valid docking simulation.^26^

#### 2.3.2 Protocol Evaluation Using Decoys Procedures

The application of validation techniques in molecular docking studies can significantly increase the reliability of virtual screening results.^27,28^ Vieira et al.^28^ evaluated the performance of scoring functions in 102 protein targets, using over 22,000 active compounds and over 1 million decoy molecules, demonstrating that the programs can discriminate up to 70% of active compounds through molecular docking. In another study, Chilingaryan et al.^29^ applied this methodology to systems with *Hs*DHODH to identify different combinations of molecular docking consensus scoring. Years later, the authors were able to demonstrate improvements in terms of recognizing potential successful compounds, which are of paramount importance in screening applications.^30^

Thus, a second docking evaluation approach was conducted using the decoy generation methodology. Initially, inhibitors of *Hs*DHODH with inhibition constant (*K*_i_) reported values were searched in ChEMBL.^31^ Compound screening was performed by removing duplicates and applying corrections for ionic regions in the SMILES code. In total, 60 compounds were identified, of which 20 were considered active (*K*_i_ between 1.7 and 21 nM) and 40 were considered inactive (*K*_i_ range of 485–90,000 nM).

The SMILES of the active compounds were used to generate decoys through the Directory of Useful Decoys-Enhanced (DUD-E) platform.^32^ A total of 51 decoys were generated for each active compound in the series. Subsequently, the three-dimensional structures of the active compounds, inactive compounds, and decoys were built using OpenBabel,^16^ applying the standard pH of 7.4, three-dimensional structure generation, and hydrogen bonds. The compounds were generated in .mol2 format for use in GOLD and in .pdbqt format for use in Autodock Vina.

After obtaining the structures of the compounds, molecular docking was performed using the prepared protein structure and the same coordinates as in the redocking process in the GOLD and Vina programs. The score acquisition was computed, and a second variable was created using a value of 1 for active compounds and a value of 0 for inactive compounds and decoys. The receiver operating characteristic (ROC) curve was used to evaluate the performance of binary diagnostic classification between active and inactive compounds, computed through the server https://stats.drugdesign.fr/.^33^ In this process, it is estimated that a higher area under the curve (AUC) indicates better discriminatory performance, where an AUC of 0.5 indicates no discrimination.^34^

#### 2.3.3 Molecular Docking Simulations

After protocol evaluations using redocking and decoy calculations, molecular docking was performed on scoring functions with AUC above 90%. Docking was carried out for the 10 compounds and the inhibitors brequinar and teriflunomide against the prepared protein structure of *Hs*DHODH as described in the redocking procedure. Ultimately, the best pose of the compounds in each scoring function was submitted to a consensus analysis using the rank-by-number strategy.^35^ In this approach, a normalization of docking scores obtained with different scoring functions is performed by dividing each score by the highest score among ligands, and then calculating the average normalized score over all scoring functions.

### 2.4 Molecular Dynamics Simulations

Molecular dynamics (MD) simulations were performed using Gromacs v. 2021.2 software^36^ on the complexes formed by *Hs*DHODH with four compounds: (i) brequinar; (ii) compounds **2d** and **2e**—the best scored ones, and (iii) **2a**, a representative low scored compound. For this purpose, the best docked pose obtained with the best predicted score was selected as initial conformation for the ligands. CHARMM36 force field^37^ parameters were calculated using CGenFF^38^ to generate the ligand and cofactor topologies. The solvent properties were emulated using the TIP3P water model with a dodecahedron box large enough to allow for a minimum of 1.0 nm space from the protein to the box walls. The system charge was neutralized with the addition of ions. Energy minimization of the solvated systems was carried out using the steepest descent algorithm for 5000 steps in order to remove steric clashes and unfavorable contacts. Subsequently, equilibration was performed in two phases. First, a 1 ns NVT ensemble simulation was conducted to stabilize the system temperature at 300 K using the velocity-rescale (V-rescale) thermostat with a coupling constant of 0.1 ps.^39^ In the second phase, a 1 ns NPT ensemble simulation was performed to equilibrate the system pressure at 1 bar using the Parrinello–Rahman barostat.^40^ During equilibration, bond lengths involving hydrogen atoms were constrained using the LINCS algorithm,^41^ allowing the use of a stable integration time step of 2 fs for the equations of motion. Long-range electrostatic interactions were treated using the particle mesh Ewald (PME) method.^42^

Production MD simulations were performed in three replicas starting from different velocities, each for a run time of 200 ns. After calculation, each trajectory was centered around the protein and visualized using UCSF Chimera. After the system reached stability, the most representative cluster of each MD trajectory was generated using the *gmx cluster* flag of Gromacs v. 2021.2. The RMSD and RMSF plots, along with standard deviation and means over the three replicas were generated and analyzed using DynamiSpectra,^43^ aiming at evaluating structural stability of the ligand–receptor complexes along the MD trajectories.

### 2.5 Calculation of Binding Energy Components (MM-PBSA)

The gmx_mmpbsa tool^44^ was employed to estimate the binding free energy (Δ*G*_binding_) of the *Hs*DHODH–ligand complexes. For this purpose, the stabilized complex obtained after MD simulations, represented by the structure (.tpr) and the trajectory files (.xtc) were utilized as input. The calculation of the binding free energy was performed using the molecular mechanics Poisson–Boltzmann surface area (MM-PBSA) method, and the free energy contribution was calculated to analyze the energetic terms and estimate the average free energy contribution of each residue. The Matplotlib in Python3 script was used to evaluate the most favorable amino acid residues.

### 2.6 Enzyme Assays

#### 2.6.1 Determination of Compound Purity by HPLC

The 6-fluoro-2-(aryl)­quinoline-4-carboxylic acid derivatives (compounds **2a**–**f**, **2j** and **2k**) under study, whose chemical structures are presented in Figure 1, were synthesized and characterized by Olegário et al.,^11^ regarding nuclear magneic resonance and high-resolution mass spectra. Herein, we also present the high-performance liquid chromatography (HPLC) analysis for purity determination. Analytical HPLC was performed on a Shimadzu Proeminence chromatograph equipped with LC-20AT binary solvent pump, SPD-M20A detector with diode array, autosampler and CBM-20A controler, using a GIST-C_18_ column (250 × 4.6 mm^2^, S-5 μm) (Shimadzu, Kyoto, Japan). Ultrapure water obtained using a Milli-Q purification system (Millipore) and HPLC-grade acetonitrile (Biograde, Brazil) were used as the mobile phase in HPLC analyses and for sample preparation. The solvents were subjected to ultrasonic cavitation using an ultrasonic washer (model Q13*L*/37, Eco-sonics) prior to their use in the HPLC system.

#### 2.6.2 Protein Production and Purification

Human DHODH (*Hs*DHODH) expression was conducted following a protocol previously established in our laboratory.^45^ The protein was expressed using *Escherichia coli* strain BL21 Codon Plus (DE3) in a rich culture medium (20 g/L tryptone, 15 g/L yeast extract, 8 g/L NaCl, 2 g/L sodium dibasic phosphate, 1 g/L potassium monobasic phosphate) supplemented with antibiotics kanamycin (30 μg/mL) and chloramphenicol (34 μg/mL). Protein expression was induced with 100 μM isopropyl-β-d-thiogalactopyranoside (IPTG) at 18 °C for 24 h. Cells were initially isolated by centrifugation and resuspended in lysis buffer (10 mM imidazole, 50 mM Tris base pH 7.5, 1% Triton X-100, 600 mM NaCl, 10% glycerol, 1 mM phenylmethylsulfonyl fluoride). The suspension was sonicated and centrifuged for 30 min at 16,000*g* before purification.

The purification was carried out by affinity chromatography on a Ni-NTA Agarose column (Qiagen Cat. No. 30210) using Buffer A (10 mM imidazole, 50 mM Tris base pH 7.5, 0.15% Triton X-100, 600 mM NaCl, 10% glycerol) and a gradient of increasing imidazole concentration up to 500 mM. The fraction containing the protein was subjected to buffer exchange using a Centricon concentrator to remove imidazole. Removal of the histidine tag was performed using ULP1 protease. After overnight incubation with ULP1 protease at 4 °C, the mixture was reapplied to the affinity column, and the protein was recovered without the histidine tag on Buffer A without imidazole. The purity of the protein was assessed by SDS-PAGE gel.

For inhibitory assays, the protein was diluted to a final concentration of 800 nM in Buffer A measured at 454 nm using the molar extinction coefficient (ε_454 nm_) of 14.26 mM^–1^ cm^–1^.

For X-ray crystallography studies, the protein purification was adapted from Lewis and co-workers, including an additional purification based on size exclusion chromatography (SEC), performed at pH 7.4 in a buffer containing 50 mM HEPES (pH 7.4), 400 mM NaCl, 10% (v/v) glycerol, 1 mM EDTA, and 0.05% (v/v) Thesit.^46^ Fractions corresponding to the homogeneous peak obtained from SEC were analyzed using SDS-PAGE, pooled, and concentrated to 30 mg/mL. Protein concentration was determined based on a theoretical extinction coefficient (ε_280 nm_) of 15.9 mM^–1^ cm^–1^ and a theoretical molecular weight of 39,887 Da.

#### 2.6.3 Inhibitory Assays

Inhibitory assays were conducted by indirect measurement of enzymatic activity, monitoring the reduction of 2,6-dichlorophenolindophenol (DCIP), according to a previously established protocol.^45^ Readings were performed on a 96-well microplate reader, in a reaction buffer containing 60 μM DCIP, 50 mM Tris pH 8.15, 150 mM KCl, 0.1% Triton X-100, 500 μM DHO, 100 μM Coenzyme-Q0, and inhibitor at varying concentrations. Inhibitors were previously diluted in 100% dimethyl sulfoxide (DMSO). All assays were conducted in a reaction medium containing 2.5% DMSO. For single-dose assays, inhibitors were used at a concentration of 250 μM, and for dose–response assays, the initial inhibitor concentration was determined for each inhibitor based on the percentage inhibition in the single-dose assay. The dose–response curve was obtained from serial dilution of compounds in the reaction buffer. The reaction was performed in triplicate, initiated by adding 195 μL of reaction buffer containing inhibitors to 5 μL of *Hs*DHODH protein solution, for a final concentration of 20 nM enzyme. As a blank, 195 μL of reaction buffer containing inhibitors was added to 5 μL of protein purification buffer. The reaction was monitored at 610 nm every 3 s over 60 s for each concentration and each compound tested. As a control, 5 μL of enzyme was added to 195 μL of 2.5% DMSO reaction medium, without the presence of inhibitors. Enzymatic velocity was calculated for each reaction, and the percentage of relative enzymatic activity was determined relative to the control (DMSO). The IC_50_ value was determined from the graph of relative enzyme activity versus the logarithm of the inhibitor concentration. The dose–response curve was fitted according to eq 1 using GraphPad Prism 5 software.

$$Y=\frac{100}{\left(1+10^{\left(X-\log\;{\mathrm{IC}}_{50}\right)}\right)}$$ 1

### 2.7 X-ray Crystallography Procedure

The methodology was adapted from Lewis and co-workers.^46^ *Hs*DHODH was incubated at 20 mg/mL with 2 mM DHO and 20.8 mM *N*,*N*-dimethyldecylamine-*N*-oxide (DDAO), and 1 mM of inhibitor for 2 h at 294 K, and crystallized by the sitting-drop vapor diffusion method at 294 K. Crystals were obtained after 7 days by mixing 1 μL of protein solution and 1 μL of crystallization condition containing 0.1 M sodium acetate trihydrate pH 4.8, 1.8–2.0 M ammonium sulfate and 30% (v/v) glycerol. Cryogenic X-ray diffraction data were collected at the beamline Manacá in CNPEM. 1800 frames were acquired with an oscillation step of 0.2° and 0.1 s of exposure and a crystal-to-detector distance of 129.52 mm. Data was processed by XDS^47^ via autoPROC. Initial phases were obtained by molecular replacement with phaser.phenix^48^ using the apo form of one DHODH structure (PDB ID 1D3G).^49^ Model building and refinement were performed with Coot^50^ and Refmac. The quality of the final models was validated by MolProbity.^51^ Figures were prepared with PyMOL (The PyMOL Molecular Graphics System, Version 4.6.0 Schrödinger, LLC.). Structures were analyzed with Coot and PyMol.

### 2.8 ADME Prediction

To analyze the pharmacokinetic profile of the 2-aryl-quinoline-4-carboxylic acid derivatives under study, in silico predictions of absorption, distribution, metabolism and elimination (ADME) parameters were calculated using the SwissADME web server.^52^ The gastrointestinal absorption (GI), Log *P*_o/w_, brain blood barrier (BBB) permeation, P-glycoprotein (Pgp) substrate binding, and drug-likeness with Lipinski’s rules were evaluated.

## 3 Results and Discussion

### 3.1 Inverse Virtual Screening Identifies HsDHODH as the Best Target

The application of target fishing methodologies has provided validation support in numerous literature reports, strengthening the role of computational target prediction and demonstrating a marked increase in its use over time.^53^ The ligand-based target fishing approaches exploiting an in-house curated library and employing three different web servers identified human DHODH (*Hs*DHODH) as the target with highest probability for compound **2d** (Figure 2, Table S1). Although TargetNet predicted multiple proteins with comparable probabilities, SwissTargetPrediction and SEA consistently ranked HsDHODH with markedly higher probabilities, supporting its identification as the primary target of compound **2d**. The ad-hoc inverse virtual screening, which structurally compared compound **2d** against a database of all PDB ligands, identified several high-scoring hits, reinforcing the findings from the web servers. This approach also identified human DHODH (*Hs*DHODH) as the most likely target. Notably, the highest similarity scores were observed with known *Hs*DHODH inhibitors, including two brequinar analogues: F51 (PDB ID 6CJG)^7^ with a PC-Score8Q of 0.912, and BRE (PDB ID 1D3G)^49^ with a score of 0.910 (Figure 2). Other potential targets were also suggested, such as tankyrase-2 (TNKS2), based on similarity to ligand 9AW (score: 0.838),^54^ and VEGFR2 kinase, indicated by ligand C52 (score: 0.812).^55^ However, the pronounced structural similarity to multiple known DHODH inhibitors provided the strongest evidence for pursuing *Hs*DHODH as the primary target.

**Figure 2:**
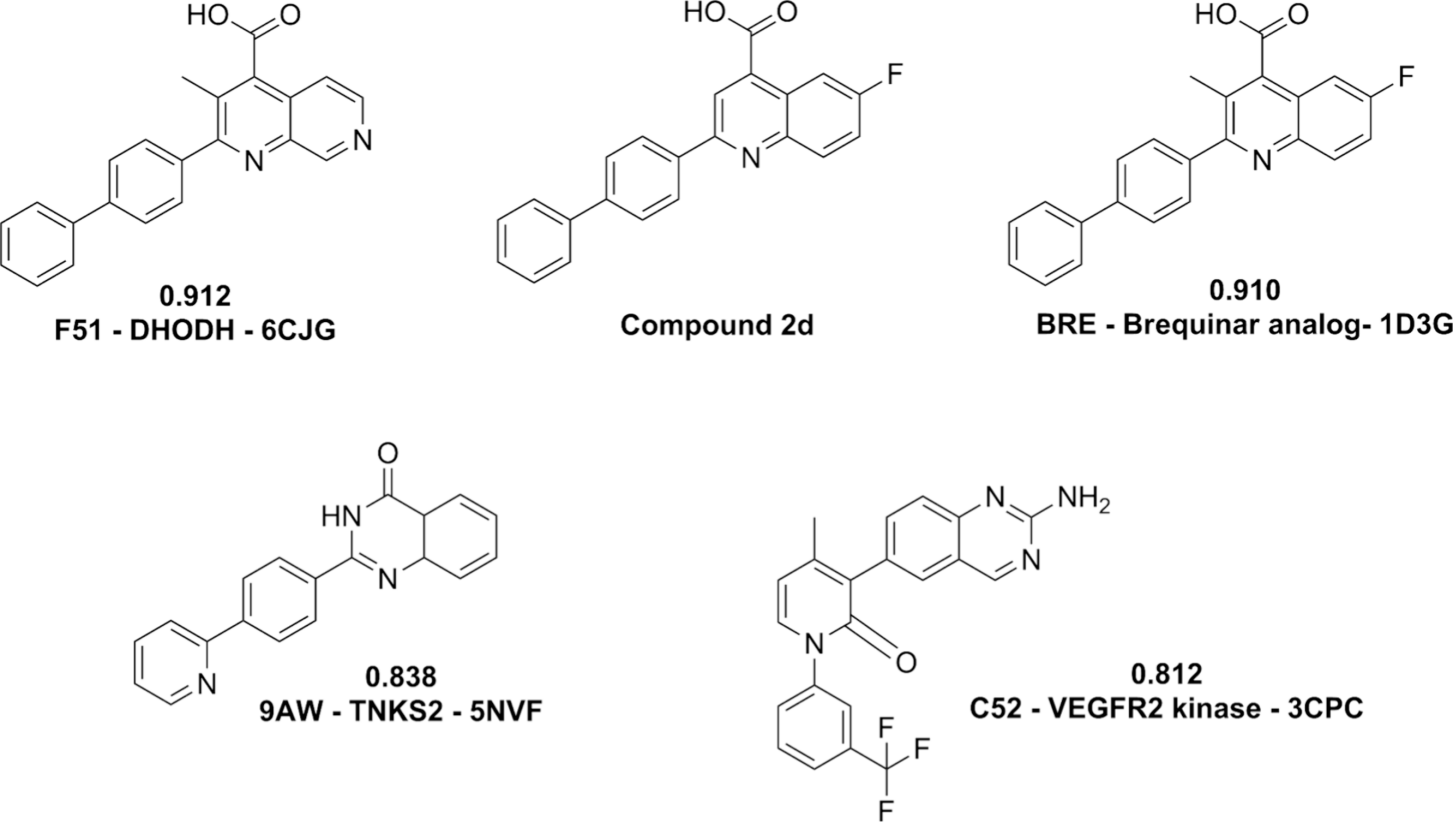
Results of the ad-hoc ligand-based inverse virtual screening for compound **2d**. The image displays the query compound (center) and the four most structurally similar ligands found in the PDB, along with their respective PC-Score8Q similarity scores and their associated protein targets and PDB entry codes.

Dihydroorotate dehydrogenase (DHODH) (EC 1.3.99.11) is a flavoenzyme that catalyzes the rate-limiting step in de novo pyrimidine biosynthesis,^56^ facilitating the reduction of the substrate l-dihydroorotate to orotate through a flavin (cofactor)-mediated reduction process. The enzyme *Hs*DHODH is involved in the pyrimidine biosynthesis pathway, enabling the production of precursors required for DNA, RNA, glycoproteins, and phospholipids.^57^ Notably, studies show its high expression in proliferative cells, which plays a significant role in tumor cell proliferation.^58^ *Hs*DHODH inhibitors have been subjected to clinical trials to evaluate its anticancer activity.^59,60^ These compounds have been demonstrated to reduce levels of pyrimidine essential for nucleotide formation.^60,61^ DHODH inhibition has been recognized as a promising strategy in cancer therapy. Its efficacy against various cancers, such as acute myelogenous leukemia,^62^ lung cancer,^63^ multiple myeloma cells,^64^ neuroblastoma cells,^65^ and breast cancer^66^ has been documented in the literature. Additionally, inhibition of *Hs*DHODH is known to decrease cellular levels of uridine monophosphate and T-cells proliferation,^67^ which has been proved to be beneficial for the treatment against autoimmune diseases. The inhibitor teriflunomide was approved for the treatment of rheumatoid and psoriatic arthritis,^68^ and also for multiple sclerosis.^69^ All together, these findings position *Hs*DHODH as a validated and versatile target with significant potential in cancer and immune-mediated disorders.

### 3.2 Molecular Docking Validation for *Hs*DHODH

Following the identification of *Hs*DHODH as a potential target for the quinoline derivatives, validation approaches of the docking protocol were executed. In the first strategy, the redocking of JTU (the brequinar analog cocrystallized with *Hs*DHODH—PDB ID 4JTU) utilizing Autodock Vina yielded an RMSD of 0.32 Å between the best docked pose and the X-ray determined conformation. Similarly, within GOLD software, the scoring functions displayed RMSD values as follows: 0.83 Å for ASP, 0.47 Å for ChemPLP, 0.22 Å for ChemScore, and 0.43 Å for GoldScore (Figure 3A).

**Figure 3:**
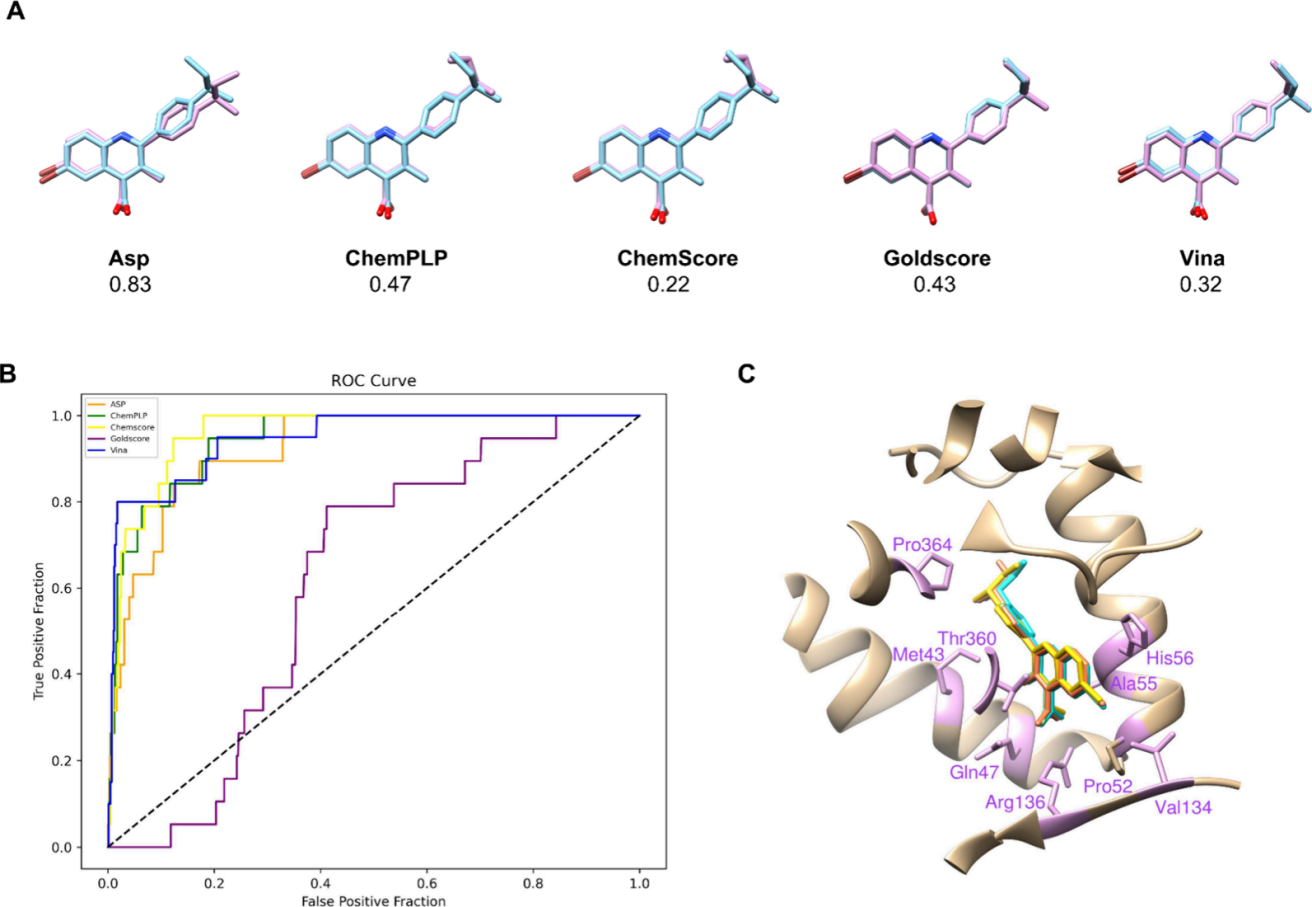
Validation results of the docking protocol for *Hs*DHODH using distinct scoring functions. (A) Overlay depicts the cocrystallized inhibitor JTU (a brequinar analogue, PDB ID 4JTU), with the X-ray pose in pink and the docked one in blue, along with RMSD values (in angstroms). (B) AUC obtained in decoy validation is highlighted in coral for ASP, golden for ChemScore, purple for GoldScore, light green for ChemPLP, and blue for Vina. (C) Interactions between the inhibitor and the amino acid residues of *Hs*DHODH are illustrated in the top 4 evaluated functions, wherein ASP is represented in coral, ChemScore in golden, ChemPLP in light green, and Vina in cyan.

Decoy validation yielded an AUC of 0.94 in Vina, while in the GOLD program, the AUC values were 0.92 for ASP, 0.95 for ChemScore, 0.61 for GoldScore, and 0.94 for ChemPLP (Figure 3B). It is noteworthy that, except for GoldScore, all functions indicated a good ability to discriminate between true binders and decoys. Since ASP, ChemScore, ChemPLP, and Vina showed predictions above 90%, they were used to perform molecular docking with the quinoline series under study (Figure 1). Additionally, it is observed that the brequinar analogue JTU (the ligand cocrystallized with *Hs*DHODH—PDB ID 4JTU) exhibited similar conformation predicted by the different scoring functions, showing hydrogen bonds between the carboxylic acid moiety and the residues Arg136 and Gln47, and hydrophobic interactions with His56, Ala55, Pro52, Val134, Met43, Thr360, and Pro364 (Figure 3C).

### 3.3 HsDHODH as the Target for Quinoline Derivatives

After molecular docking validation, the series of 6-fluoro-2-phenylquinoline-4-carboxylic acid derivatives, and also the reference compounds JTU, brequinar and teriflunomide, were assessed for interactions with the *Hs*DHODH binding site utilizing the top four validated scoring functions: ASP, ChemScore, ChemPLP, and Vina. Our findings, shown in Table S2, revealed that the derivatives exhibited scores close to JTU (the brequinar analogue cocrystallized with *Hs*DHODH—PDB ID 4JTU). Subsequent consensus analysis identified compound **2d** as yielding the most favorable results, followed by compound **2e**. Compound **2a** was selected as a negative control based on its low docking scores, indicating reduced affinity for the active site of *Hs*DHODH. Among the evaluated compounds, **2a** showed lower score values across the scoring functions analyzed: Asp (36.04), Chemscore (37.75), ChemPLP (73.62), and Vina (−9.57). Additionally, its substituent group is structurally simple, consisting of only a single benzene ring, which limits the potential for specific interactions with enzyme residues.

The interaction of these compounds with the *Hs*DHODH binding site reveals an overlap of the quinoline core with brequinar, suggesting potential mimicry of its biological activity (Figure 4). In terms of interactions with the binding site, compounds **2d** and **2e** display π interactions between the quinoline ring and the residues Met43, Pro52, Val134, Ala55, and His56, similarly to brequinar. Additionally, the biphenyl group engages in π interactions with residue Pro364, whereas this interaction is absent in the naphthyl (**2e**) and benzene (**2a**) group. Furthermore, hydrogen bonds are established between the ketonic portion of the quinoline ring and the residues Arg136 and Gln47. Similarly to those found in other *Hs*DHODH inhibitors,^7^ all quinolinic derivatives display π-type interactions with the cofactor flavin mononucleotide (FMN).

**Figure 4:**
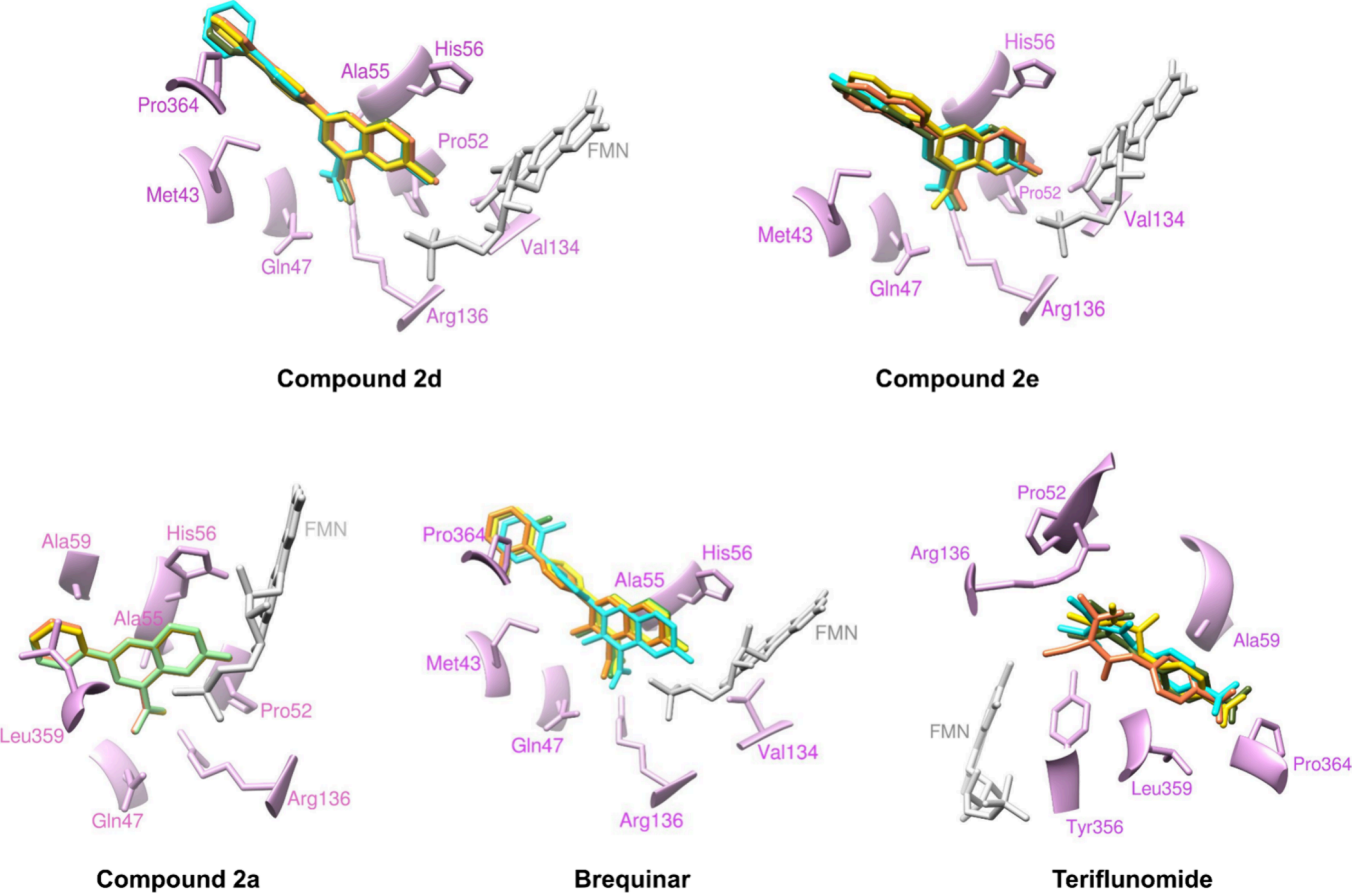
Molecular docking poses of representative and reference compounds obtained with ASP (coral), ChemScore (golden), ChemPLP (light green), and Vina (cyan) scoring functions at the *Hs*DHODH enzyme. Overlay of the best pose for compounds **2d**, **2e**, **2a**, brequinar and teriflunomide.

Brequinar was discovered to be one of the strongest known inhibitors of *Hs*DHODH.^59^ Thus, inhibitors with structures analogous to brequinar have been reported in the literature and have been shown to be responsible for inhibiting tumor growth in in vivo studies, and sensitizing cells to apoptosis.^70^ On the other hand, teriflunomide, also known as A771726, is the active metabolite of leflunomide and binds *Hs*DHODH at the same site as brequinar. In vivo studies have demonstrated its ability to inhibit adhesion molecules, cytokines, protein tyrosine kinases, nuclear factor-kB (NF-kB) activation, and cyclooxygenase 2 activity, exerting an antiproliferative effect.^71^ The interactions with *Hs*DHODH are promoted through hydrogen bonds with Arg136 and Tyr356, while π interactions are formed with Leu359, Pro364, Ala59, and Pro52. These interactions were confirmed by X-ray crystallography,^49^ where the aromatic portion of the trifluoromethyl ring establishes numerous hydrophobic contacts with the active site. The authors also highlight that there are fewer inhibitor–protein interactions in the teriflunomide complex compared to the brequinar complex due to the smaller size of teriflunomide, which is less ordered than in the brequinar complex.

### 3.4 Stability of Quinolinic Derivatives with HsDHODH

The structural stability of the ligand–receptor complexes was evaluated through 200 ns molecular dynamics simulations performed in triplicate. All reported mean and standard deviation values correspond to averages calculated across the three independent 200 ns replicas. Analysis of the protein backbone RMSD (Figure S1A,B) highlights distinct stabilization behaviors among the *Hs*DHODH complexes. The brequinar system reaches a stable RMSD earlier, between 20 and 30 ns, and maintains the lowest fluctuation amplitude throughout the simulation, consistent with a more rigid and well-defined protein–ligand complex. In contrast, the complexes with **2d** and **2e** exhibit a more gradual initial relaxation and show comparable backbone RMSD ranges over the remainder of the trajectories, with some variability across the three replicas, but no evidence of progressive structural drift. These results indicate that both **2d** and **2e** support a globally stable protein conformation over 200 ns, albeit with slightly higher flexibility than observed for brequinar. By comparison, the **2a** complex displays the largest RMSD values and fluctuations, reflecting increased conformational mobility of the protein in this system.

Analysis of the ligand RMSDs (Figure S1C,D) demonstrated that brequinar exhibits a stable binding pose from the beginning of the simulation, with consistently low RMSD values, indicating immediate and persistent accommodation within the binding site. In contrast, the quinoline derivatives **2d** and **2e** display an initial adjustment period (at approximately 15–20 ns), after which their RMSD values decrease, reflecting progressive accommodation of these ligands within the binding pocket. Compound **2a** shows a distinct behavior, with an increase after approximately 17 ns, indicating a greater positional mobility within the binding site. The reference to 17 ns therefore marks the accommodation of **2d** and **2e** and, simultaneously, the onset of increased mobility for **2a**, rather than global protein backbone convergence. Once stabilized, **2d** and **2e** remain well accommodated for the remainder of the trajectories, whereas **2a** displays continued fluctuations consistent with its reduced interaction persistence.

Accordingly, RMSF analyses (Figure S2A,B) indicated limited residue mobility within the binding region, with fluctuations below 1.5 Å, further supporting complex stability. These findings are in agreement with previous reports on *Hs*DHODH complexes with brequinar and leflunomide, which also displayed low dynamic variability and well-converged trajectories.

The interactions of the protein–ligand complex are shown in Figure 5 for the most representative cluster, especially by hydrogen bonds between the quinoline group and residues Gln47, Arg136 and Thr360. In all ligands, hydrophobic residues such as Ala55, Ala59, Leu46, Pro364, and Phe62 were observed participating through π–alkyl or π–π T-shaped interactions, suggesting that this hydrophobic portion may play an important role in stabilizing the compounds. In addition, residue Arg136 engages in hydrogen bonding, also mediated by water molecules. This conservation indicates that the region near Arg136 may serve as a pharmacophoric anchoring point. In some cases, structural water molecules participate as hydrogen bond bridges, connecting polar groups of the ligands to the active site. These features were also found in the crystallographic structure, underscoring their essential role in biological activity.

**Figure 5:**
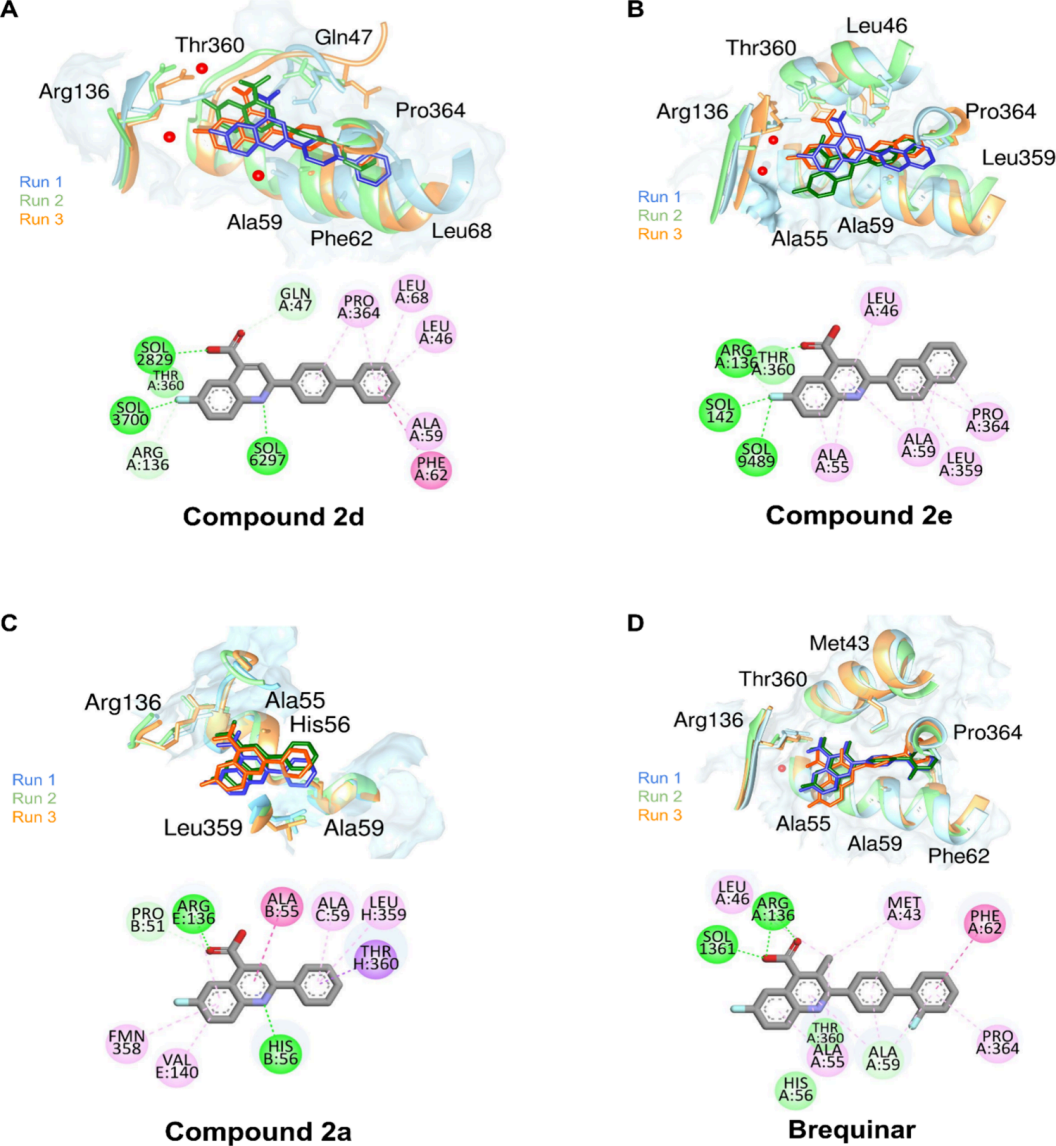
Superimposition of the structures of the most populated cluster of the triplicate molecular dynamics simulations (run 1 in blue, run 2 in green and run 3 in orange), where water molecules are represented by red balls. In the 2D representation, the ball representation of the amino acid residues highlights the types of interactions, as follows: green for hydrogen bonds, purple for π–σ, dark pink for π–π stacked, and light pink for π–alkyl.

Compound **2d**, which showed the best interaction profile, combines multiple hydrogen bonds, including water-mediated ones (SOL2829, SOL3700, and SOL6297), and hydrophobic contacts with Leu46, Ala59, and Phe62, in addition to a halogen interaction involving fluorine. This combination may suggest greater stability and complementarity in the active site. By contrast, brequinar maintained hydrogen bonding with Arg136, explored the conserved hydrophobic core (Ala55, Ala59, Leu46, Pro364, Phe62), and displayed a structural water molecule SOL1361 contributing to the stabilization of its carbonyl group (Figure 5).

For compound **2e**, the results were similar, with notable π–sulfur interaction with Met43 and water-mediated bonds (SOL142 and SOL9489), which expand the range of contacts. Finally, compound **2a** was the least active, establishing direct interactions with His56 and Tyr317 and a π–σ contact with Thr360 and Gly324, but with no significant participation of water molecules and a lower diversity of stabilizing interactions, which may explain its reduced affinity (Figure 5).

Combining these findings with the analysis of MD trajectories reveals that water molecules play distinct and functionally relevant roles in stabilizing the *Hs*DHODH–ligand complexes (Figure 5). Specifically comparing systems *Hs*DHODH–**2d** and −brequinar, a conserved polar region near Arg136 and Thr360 accommodates structured water molecules that mediate hydrogen-bond networks between the ligand carboxylate or heteroatoms and the protein. For compound **2d**, stabilization relies heavily on a solvent-assisted anchoring mechanism, in which recurrent water bridges connect the quinoline scaffold to Arg136 and neighboring residues. These water-mediated interactions compensate for the reduced number of persistent direct hydrogen bonds and enable stable binding while allowing increased conformational adaptability.

In contrast, brequinar displays a more rigid binding mode dominated by direct and persistent protein–ligand interactions, supplemented by a single, well-ordered structural water molecule that consistently bridges its carbonyl group to the binding site. This denser and more localized interaction network contributes to the earlier equilibration and reduced backbone fluctuations observed for the brequinar complex. Altogether, these results indicate that ordered water molecules are crucial for ligand recognition in this system, with different functional roles: for brequinar, a single water molecule reinforces a rigid, highly stabilized complex, while for **2d**, water molecules enable dynamic but consistent binding, which reflects in its high enzymatic affinity.

The residue contribution analysis (Figure S3) provided by the MM-PBSA approach showed the binding site residues contributing for the formation of interactions, mainly Arg136 and Pro364, with energies close to 10 kcal/mol, followed by Thr360 close to 8 kcal/mol. Among the quinolinic derivatives studied, we observe that compound **2d** exhibited the lowest average of binding free energy in the triplicate simulations, indicating an affinity to *Hs*DHODH higher than that of brequinar (Table 1), which corroborates the molecular docking predictions.

**Table 1:** MM-PBSA Results for Each Triplicate and Average Δ*G* Estimation for the Simulations Undertaken for the Complexes Formed by *Hs*DHODH and Brequinar, Compounds **2d**, **2e**, and **2a**

	binding energy (kcal/mol)			
*Hs*DHODH– ligand complex	run 1	run 2	run 3	(kcal/mol)
brequinar	–46.65	–46.52	–42.56	–45.24
**2d**	–49.04	–49.49	–46.95	–48.49
**2e**	–44.69	–45.00	–44.34	–44.67
**2a**	–38.76	–38.29	–38.20	–38.41

These results demonstrated that compound **2d** exhibited the most favorable interaction profile due to a combination of hydrogen bonds, including those mediated by water molecules, as well as hydrophobic contacts with conserved residues (Leu46, Ala59, Phe62). This pattern resembles that described for brequinar in the study by Khairy et al.,^23^ where 100 ns molecular dynamics simulations revealed the stability of the brequinar–*Hs*DHODH complex, sustained by persistent interactions with Arg136, Ala55, Ala59, Leu46, Phe62, and Pro364, amino acid residues also found in our research. The authors also reported that some natural inhibitors tested (such as silibinin) shared key interactions with brequinar, highlighting the relevance of residues such as Arg136, Phe62, and Ala59 in complex stabilization. The convergence of these findings with our results reinforces the importance of the conserved hydrophobic core and the mediating role of water as critical elements for the affinity and selectivity of *Hs*DHODH inhibitors.

It should be noted that MM-PBSA calculations were employed here primarily as a qualitative tool to analyze residue contributions and general energetic trends within the binding site rather than as a rigorous method for ranking binding affinities among closely related ligands. End-state methods such as MM-PBSA can provide useful insights into interaction patterns but are known to have limited quantitative accuracy when discriminating between compounds within the same chemical series. In the present study, the relative potencies of the compounds are therefore primarily supported by the experimental enzymatic inhibition data (Section 3.6), while the MM-PBSA analysis is used to complement the structural interpretation of the molecular dynamics simulations and to highlight the key residues contributing to ligand binding.

The native contacts of each ligand were analyzed using CPPTRAJ,^72^ with an interaction cutoff distance set at 4.0 Å. Persistent contacts (defined as occupancy ≥10%) of ligand interactions with active-site residues are summarized in Table 2, where it is possible to notice a predominance of contacts with Thr360 in all ligands. Additionally, hydrogen bonds were quantified applying a distance threshold of 3.5 Å between donor and acceptor atoms (shown in Table S3).

**Table 2:** Percentage of Native Contacts with Binding Site Residues (4.0 Å Cutoff)

**compound**	**residue**	**%**
**2a**	THR360	22.15
	ALA59	15.67
	ARG136	14.95
**2e**	THR360	24.05
	PRO364	17.54
	ALA59	15.41
**2d**	THR360	14.73
brequinar	ALA59	26.70
	THR360	26.04
	ARG136	20.72
	HIS56	18.13
	ALA55	16.73
	PRO364	13.47

The analysis of native contacts and hydrogen bond occupancies revealed clear differences among the ligands. Brequinar maintained highly persistent direct hydrogen bonds with Arg136 and Thr360 and exhibited the highest fraction of native contacts, particularly with Ala59, Thr360, and Arg136, consistent with a stable binding mode. Compounds **2a**, **2d**, and **2e** displayed fewer persistent hydrogen bonds and generally lower native contact fractions, indicating more dynamic interactions within the binding pocket. Interestingly, molecular dynamics simulations showed that both brequinar and **2d** can establish water-mediated hydrogen bonds with Arg136; however, **2d** relied on a larger number of bridging water molecules (three, compared to only one in brequinar), suggesting a more solvent-assisted binding mode. These observations are consistent with the MM-PBSA per-residue decomposition, which highlighted Arg136 and Thr360 as major contributors for all ligands, but showed a broader distribution of stabilizing interactions for brequinar compared to the narrower and more fluctuating profile of **2d**.

### 3.5 X-ray Results and Binding Mode Analysis

X-ray crystallography experiments were performed to validate the predicted binding modes of compounds **2d** and **2a** within the HsDHODH binding site and to compare them with the well-characterized binding profile of brequinar. The complexes of *Hs*DHODH with **2d** and **2a** were resolved at 1.51 and 1.34 Å resolution, respectively. The final coordinates and structure factors have been deposited in the Protein Data Bank under accession codes 9XYG and 9XYF. Details for data collection and statistical results for data processing are presented in Table S4, while results for structure refinement are shown in Table S5.

Overall, the structures present the characteristic α–β barrel fold typical of *Hs*DHODH structures (Figure S4a). The structures are very similar to each other (Figure S4a,b), with a mean root-mean-square deviation (RMSD) of 0.081 Å. The structures of *Hs*DHODH in complex with **2a** span 363 residues from Asp34 to Arg396, and **2d** span 365 residues from Thr32 to Arg396.

The structures contain FMN and ORO as well as their respective inhibitors. The *Hs*DHODH–**2a** structure contains 312 water molecules treated as oxygens, 4 sulfate ions, 3 acetate ions, 1 glycerol molecule, and 2 chlorine ions. In contrast, the *Hs*DHODH–**2d** structure contains 267 waters, 3 sulfate ions, 3 chlorine ions, and a decylamine-*N*,*N*-dimethyl-*N*-oxide molecule. The refined electronic maps show a well-defined density for each inhibitor, shown in Figure S4c,d.

To evaluate the accuracy of the docking predictions, the superimposition of compounds **2d** and **2a** cocrystallized with *Hs*DHODH to the docked poses obtained with the best scoring function (Chemscore) is shown in Figure 6. This analysis reveals an excellent agreement between predicted and experimental poses, with RMSD values of 1.342 Å for **2d** and 0.762 Å for **2a**. Regarding the residues involved in ligand interactions, low RMSD values were observed, ranging from 0.060 to 0.254 Å for compound **2d**, and 0.088 to 0.294 Å for compound **2a**.

**Figure 6:**
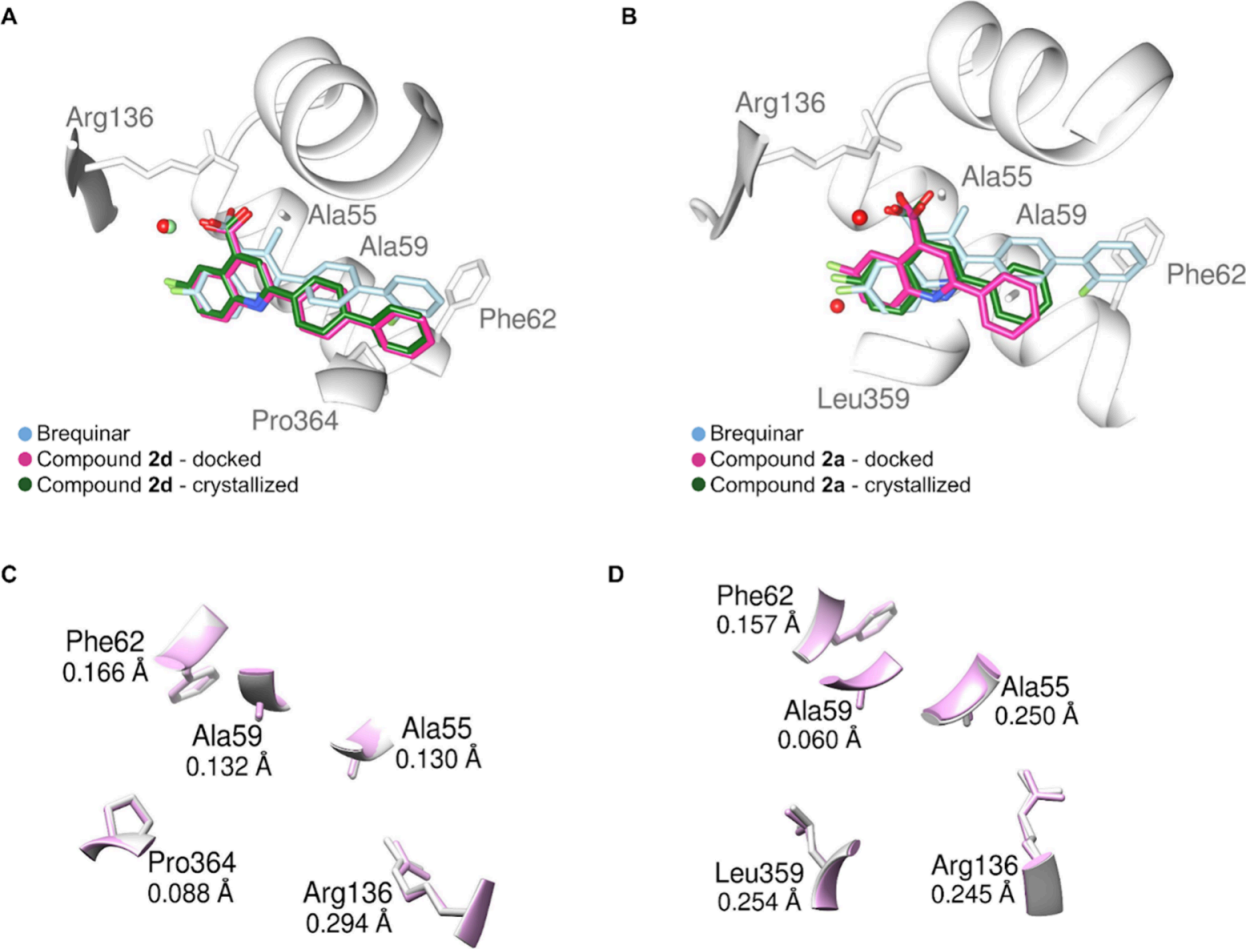
Superimposition of brequinar (blue), docked (pink) and cocrystallized (green) (A) **2a** and (B) **2d** derivatives. (C) shows the overlay of the binding site residues for the **2a**–*Hs*DHODH complex, and (D) shows the overlay for the **2d**–*Hs*DHODH complex, with the docked structures displayed in pink and the cocrystallized residues in light gray. The RMSD values (in angstroms) between the structures are highlighted. The water molecules are represented as spheres, with the crystallographic ones colored red and the molecular dynamics ones colored green.

Notably, the presence of structural water molecules interacting with the carboxylic acid of the ligands was observed in the crystal structures, as predicted by the MD simulations. The functional relevance of the water-mediated interactions observed in the simulations is supported by their correspondence with experimentally resolved water positions in the X-ray structures of **2d** and **2a** (Figure 6). As mentioned in Section 3.4, in the MD trajectories, structured water molecules consistently occupy a conserved polar region adjacent to Arg136 and Thr360, where they act as bridging elements between the ligand carboxylate/heteroatoms and the protein, thereby stabilizing ligand orientation within the binding pocket. For compound **2d**, these waters form recurrent hydrogen-bond networks that maintain the correct positioning of the quinoline core despite the reduced number of persistent direct protein–ligand contacts, effectively compensating through a solvent-mediated stabilization mechanism. Importantly, the X-ray cocrystal structure of the **2d**–*Hs*DHODH complex reveals ordered water molecules in the same region, bridging the ligand to Arg136 and neighboring residues, indicating that the water-mediated interactions observed in MD are not transient artifacts but reflect structurally relevant features of the binding mode.

Additionally, a structural comparison between the structure of brequinar docked to *Hs*DHODH and the crystallographic poses of compounds **2d** and **2a** revealed a highly similar binding orientation within the binding site, highlighting conserved interactions and alignment of key pharmacophoric features, such as Ala55, Ala59, Arg136 and Leu359.^7^ To the best of our knowledge, no X-ray crystal structure of brequinar in complex with *Hs*DHODH is currently available in the Protein Data Bank, which prevents a direct comparison for this ligand.

Importantly, while quinoline-4-carboxylic acid derivatives related to this scaffold have been previously reported as DHODH inhibitors,^10^ the present crystallographic structures provide direct structural evidence of their binding mode within *Hs*DHODH and reveal how solvent-mediated interactions contribute to the stabilization of simplified analogues such as compound **2d**.

### 3.6 Determination of Activity in HsDHODH

Prior to biological evaluation, the purity of all synthesized compounds was confirmed by high-performance liquid chromatography (HPLC). Compounds were analyzed by HPLC using a C_18_ column and a gradient elution system composed of H_2_O (A): ACN (B) with elution profile = 0.00–20.00 min (5–100% B); 20.00–25.00 min (100% B); 25.00–30.00 min (100–5% B); 30.00–35.00 min (5% B); volume injection 5 μL and flow rate of 1.0 mL/min. Retention time and purity of compounds **2a**–**f**, **2j** and **2k** are presented in Table S6, where the purity of all compounds was confirmed to be higher than 95%. The corresponding chromatograms are provided in the Supporting Information (Figures S5–S12).

To determine the enzyme inhibitory activity of the designed compounds, the DHODH biochemical assay was used. Inhibitory assays were conducted by indirect measurement of enzymatic activity, monitoring the reduction of 2,6-dichlorophenolindophenol (DCIP), according to a previously established protocol.^41^ The results of inhibitory activity expressed as IC_50_ values are summarized in Table 3. Among all quinoline-4-carboxylic acid derivatives tested, only **2d** exhibited a comparable IC_50_ value with brequinar, used as a reference compound. The derivative **2d** also was identified as the most potent inhibitor with an IC_50_ value of 27 ± 1 nM in contrast with 29 ± 1 nM for brequinar. Compound **2e** bearing a naphthyl unit linked to the quinoline scaffold and **2f** containing a *p*-fluorophenyl moiety were found to be good inhibitors with IC_50_ = 0.46 ± 0.02 and 0.92 ± 0.04 μM, respectively.

**Table 3:** Experimental Results of Enzymatic Inhibition of the 6-Fluoro-2-phenylquinoline-4-carboxylic Acid Derivatives against the *Hs*DHODH Enzyme

**compound**	**single dose** *Hs* **DHODH activity (%)** ^a^ ^,^ ^b^	**IC**_**50**_ **(μM)** ^a^
brequinar	0.97 ± 0.05	0.029 ± 0.001
teriflunomide	1.03 ± 0.05	0.26 ± 0.01
**2a**	2.00 ± 0.05	1.83 ± 0.07
**2b**	0.42 ± 0.04	1.70 ± 0.08
**2c**	5.9 ± 0.3	1.5 ± 0.1
**2d**	2.0 ± 0.2	0.027 ± 0.001
**2e**	1.3 ± 0.1	0.46 ± 0.02
**2f**	1.2 ± 0.1	0.92 ± 0.04
**2j**	0.8 ± 0.2	1.14 ± 0.05
**2k**	1.3 ± 0.1	2.7 ± 0.1

a Experiments performed with ligand at 250 μM.

b Values expressed as mean ± SD.

Comparing these findings to the computational insights, it is worth noting that, although **2d** exhibited fewer persistent native contacts and did not retain the strong direct hydrogen bonds with Arg136 and Thr360 found for brequinar, its affinity to *Hs*DHODH was comparable. In brequinar, in addition to the highly persistent direct H-bonds to Arg136 and Thr360, resulting in a stable interaction network, the fluorine atom in the biphenyl moiety engages hydrophobic interactions with Ala59. By contrast, **2d** lacks this fluorine and therefore shows greater conformational flexibility of the biphenyl group, with fewer direct polar contacts. The X-ray structure of the **2d**–*Hs*DHODH complex, together with the MD simulations, revealed that this ligand compensates by recruiting multiple bridging water molecules (three waters, compared to only one in brequinar) that mediate interactions with Arg136 and neighboring residues.

Thus, while brequinar stabilizes binding through a dense network of direct interactions supplemented by a single water bridge, **2d** relies more heavily on solvent-assisted anchoring and conformational adaptability to achieve a similar net binding affinity. This illustrates how equivalent potency can arise from distinct binding strategies, highlighting the role of ordered water molecules as integral contributors to ligand recognition.

### 3.7 Lipophilicity of Quinoline Derivatives

Despite brequinar and its analogs showing excellent preclinical data, they exhibit poor water solubility and molecular aggregation at high concentrations.^73^ The prediction of Log *P* can estimate the distribution of chemical substances between hydrophilic (water) and hydrophobic (octanol) phases. Using the consensus Log *P*_o/w_ analysis in SwissADME, we observed that compound **2a**, **2d** and **2e** has a lower Log *P* than brequinar, suggesting that these compounds have a favorable solubility profile (Table 4).

**Table 4:** ADME Prediction of Compounds **2a**, **2d**, **2e** and Brequinar, Showing Gastrointestinal Absorption (GI), Log *P*_**o/w**_, No P-Glycoprotein (Pgp) Substrate, and Lipinski Rules

**compounds**	**GI**	**Log *P***_**o/w**_	**Pgp**	**Lipinski**
**2a**	high	3.12	no	no violation
**2d**	high	4.42	no	no violation
**2e**	high	4.05	no	no violation
brequinar	high	5.09	no	no violation

Inhibitors structurally related to brequinar have been widely explored due to their ability to target *Hs*DHODH, a key enzyme in pyrimidine biosynthesis with relevance in cancer and autoimmune diseases. These compounds are typically developed based on the well-established binding mode of brequinar, using a combination of computational modeling, structural biology, and biochemical assays to improve their potency and pharmacological profiles.^58^

Chen et al.^59^ identified that compounds bearing the biphenyl or naphthyl group (bulky hydrophobic) at R2, methyl substitution at R3, and carboxylic acid at R4 were the most effective inhibitors. Substituents at position R6 demonstrated that halogen substitutions provide good inhibitory activity, which decreases with decreasing electronegativity. The researchers also found that substituting the quinoline ring with a pyridine ring does not promote inhibition of *Hs*DHODH, highlighting the importance of the group. Vyas et al.^74^ replaced groups at position R2 with alkyl and steric bulky groups, obtaining inhibitory activities for the compounds thus formed. However, the results were inferior to those by Wang et al.,^19^ who found similar results when evaluating a series of quinoline derivatives as inhibitors against *Hs*DHODH. The most active compound in the series exhibited an IC_50_ of 9.7 nM and was crystallized with the enzyme. Additionally, the authors identified that a bromine at R6 and a para-alkyl substituted phenyl group at R2 are beneficial for the activity.

Addressing the poor water solubility and tendency of brequinar to form aggregates at high concentrations,^75^ Madak et al.^7^ explored the use of a 1,7-naphthyridine ring to improve solubility relative to the quinoline scaffold in newly designed *Hs*DHODH inhibitors. Although this substitution successfully enhanced aqueous solubility, it did not lead to higher potency than brequinar. Petrović et al.^9^ investigated quinoline-4-carboxylic acids as *Hs*DHODH inhibitors, focusing on aliphatic substitutions at the R2 position of the phenyl ring. Their most active compound showed an IC_50_ of 100 nM. In a later study, the same group synthesized 14 new derivatives, six of which displayed stronger inhibitory activity than the reference drug leflunomide.^76^ These compounds, with IC_50_ values ranging from 0.12 to 0.14 μM, showed cytotoxic effects against human breast cancer cell lines. Notably, the presence of halogen substituents on aromatic rings was associated with improved biological profiles.

Our findings suggest that the structural simplification of brequinar appears to be a good strategy, since the 6-fluoro-2-(aryl)­quinoline-4-carboxylic acid derivatives studied here, particularly compounds **2d** and **2e**, maintain potent *Hs*DHODH inhibitory activity while exhibiting lower predicted Log *P* values compared to brequinar, indicating a more favorable solubility profile. These results reinforce the potential of this chemical scaffold as a promising platform for the development of DHODH inhibitors with improved drug-like properties. Further optimization of this series may contribute to overcoming the solubility and bioavailability challenges commonly associated with brequinar analogs, advancing the pursuit of more effective therapeutic agents.

Indeed, the polypharmacological nature of quinoline scaffolds, while advantageous in some therapeutic settings, also implies a risk of off-target effects that must be addressed in the development of DHODH-focused inhibitors. Previous studies have shown that quinoline derivatives can interact with receptor tyrosine kinases and other signaling proteins involved in proliferative pathways, suggesting that kinase cross-reactivity represents a plausible off-target liability for this chemical class.^77^

In this context, the strong structural and energetic agreement observed here between compound **2d** and brequinar argues for a well-defined and specific binding mode within the *Hs*DHODH pocket. Nevertheless, a comprehensive assessment of selectivity will require additional studies, such as expanded in silico target profiling, comparative docking or binding free-energy calculations against representative enzyme panels, and experimental assays. Future optimization strategies may also include fine-tuning steric bulk and electronic properties at the C-2 aryl substituent and modulation of lipophilicity, aiming to preserve DHODH potency while disfavoring interactions with unrelated targets. Together, these approaches will be essential to mitigate potential off-target liabilities and to advance this chemotype toward safer and more selective DHODH-directed therapeutics.

## 4 Conclusions

In this study, we employed ligand-based inverse virtual screening to confirm human dihydroorotate dehydrogenase (*Hs*DHODH) as the highest affinity molecular target for a series of 6-fluoro-2-(aryl)­quinoline-4-carboxylic acid derivatives. Among these, compound **2d** stood out as the most potent inhibitor, exhibiting an IC_50_ of 27 ± 1 nM, comparable to the reference inhibitor brequinar (29 ± 1 nM). Docking and molecular dynamics simulations revealed stable and high-affinity interactions of compounds **2d** and **2e** within the *Hs*DHODH binding site, closely mirroring the dynamic profile of brequinar. These results were further validated by X-ray crystallography, which confirmed the binding mode of the compounds within the binding site. Notably, although the absence of fluorine in **2d** abolishes the contact with Ala59 observed for brequinar, this is offset by increased conformational flexibility that enables dynamic water-mediated interactions, maintaining high affinity. In addition, compound **2d** demonstrated a more favorable Log *P* value than brequinar, indicating improved physicochemical properties that may translate into better drug-likeness. Collectively, these findings highlight compound **2d** as a promising lead for the development of *Hs*DHODH inhibitors and underscore the value of computational target profiling and structural validation in the optimization of ligands for known druggable targets.

## Supplementary Material



## Data Availability

The data underlying this study are openly available in: (1) the published article (molecular structures, activities and properties) and its Supporting Information; (2) GitHub at https://github.com/viana1jess/JCIM_hsDHODH (machine-readable molecular structure files, topology and parameter files, simulation inputs including all simulation parameters and the random number generator seed values used for the replicas, clustered structures from each replica, ROC-curve validation data extracted from ChEMBL and DUD-E platforms, and the script for Inverse Virtual Screening); (3) RCSB Protein Data Bank (https://www.rcsb.org/) under Accession Codes 9XYG and 9XYF (X-ray crystal structures of ligand-protein complexes).

## Notes

List of predicted targets; docking scores; percentage of hydrogen bonds; details for X-ray data collection; results for structure refinement; retention time and purity of the compounds in HPLC; molecular dynamics structural analysis; contributions of residues to binding energy; cartoon representation of the complexes obtained by crystallography; and HPLC chromatograms for all compounds under study (PDF)
