# Supplementary material for: Structural Basis for Potent Inhibition of Human DHODH by Quinoline-4-carboxylic Acid Derivatives
Source: ACS Omega. 2026 Apr 27;11(18):26569–83. doi: 10.1021/acsomega.5c12978 (PMC13176990; doi:10.1021/acsomega.5c12978)

Supporting Information

Structural Basis for Potent Inhibition of Human DHODH by Quinoline-4-carboxylic Acid Derivatives

Jéssika de Oliveira Viana[a], Tayná Rodrigues Olegário[b], Marília Cecília da Silva[a], Rodrigo Cristiano[b], Euzébio Guimarães Barbosa[c], Aline Dias da Purificação[d], Marina Sena Mendes[d], Diogo Boerin[d], Maria Cristina Nonato[d], Karen Cacilda Weber[a], Claudio Gabriel Lima-Junior[b*]*

[a] Computational Quantum Chemistry Laboratory, Department of Chemistry, Federal University of Paraíba, João Pessoa, 58051-900, Paraíba, Brazil

[b] Medicinal Organic Synthesis Laboratory of Paraíba (LASOM-PB), Department of Chemistry, Federal University of Paraíba, João Pessoa, 58051-900, Paraíba, Brazil

[c] Computational Pharmaceutical Chemistry Laboratory, Faculty of Pharmacy, Federal University of Rio Grande do Norte, Natal, 59078-970, Rio Grande do Norte, Brazil

[d] Center for the Research and Advancement of Fragments and Molecular Targets (CRAFT), School of Pharmaceutical Sciences of Ribeirão Preto, University of São Paulo, Ribeirão Preto, 14040-903, São Paulo, Brazil.

* corresponding authors: karen@quimica.ufpb.br, claudio@quimica.ufpb.br

Table S1. Targets predicted by the different target fishing web-servers for compound **2d**.

SwissTargetPrediction		
**Target**	**Probability**	**Species**
DHODH	0.719000000000	*Homo sapiens*
PDE10A	0.109339753231	*Homo sapiens*
ALOX5	0.109339753231	*Homo sapiens*
PTGES	0.109339753231	*Homo sapiens*
PLA2G2A	0.109339753231	*Homo sapiens*
PPARA	0.109339753231	*Homo sapiens*
KDM4E	0.109339753231	*Homo sapiens*
MARS	0.109339753231	*Homo sapiens*
KDM4C	0.109339753231	*Homo sapiens*
CMA1	0.109339753231	*Homo sapiens*

SEA			
**Target**	**MaxTC**	**P-Value**	**Species**
DHODH	1.0	2.411e-169	*Homo sapiens*
PRS	0.59	6.91e-135	Eukaryotes/canax
MT-CO2	0.52	3.562e-102	Eukaryotes/sheep
EIF4E	0.39	1.114e-90	*Homo sapiens*
fabH	0.36	2.369e-56	Bacteria
MT-CO1	0.42	1.184e-50	Eukaryotes/sheep
PRKAB2	0.31	1.736e-47	*Homo sapiens*
EPRS	0.38	8.536e-46	*Homo sapiens*
UBE2I	0.35	7.011e-45	*Homo sapiens*
TACR3	0.46	5.614e-41	*Homo sapiens*

TargetNet		
**Target**	**Probability**	**Species**
p21-Rac1	1.0	*Mus musculus*
TUBB2B	1.0	*Bos taurus*
DUSP3	1.0	*Homo sapiens*
RELA	1.0	*Homo sapiens*
DHODH	1.0	*Homo sapiens*
MIF	1.0	*Homo sapiens*

GSK3A	1.0	*Homo sapiens*
CES1	1.0	*Homo sapiens*
PTGES	1.0	*Homo sapiens*
NR2E3	1.0	*Homo sapiens*

Table S2. Results of molecular docking scores of the quinoline derivatives on the *Hs*DHODH enzyme. The scoring values for Vina are in kcal/mol.

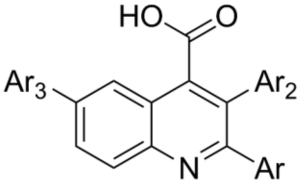

Comp.	-Ar	-Ar2	-Ar3	ASP	ChemScore	ChemPLP	Vina
2a	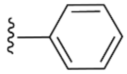	-	—F	36.04	37.75	73.62	-9.57
2b	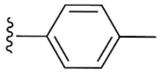	-	—F	37.23	39.25	74.89	-9.90
2c	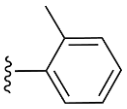	-	—F	36.99	38.43	78.29	-10.46
2d	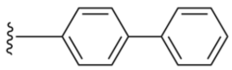	-	—F	42.18	44.37	87.34	-11.48
2e	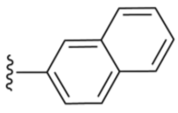	-	—F	41.20	43.59	80.32	-10.80

2f	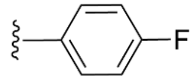	-	—F	37.94	37.47	75.50	-9.87
2j	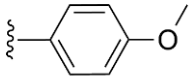	-	—F	37.50	38.15	76.53	-9.68
2k	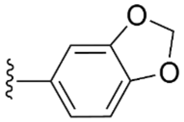	-	—F	38.86	37.84	76.27	-10.32
JTU	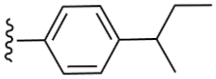	—CH_3	—Br	39.97	38.40	97.89	-10.11
Brequinar	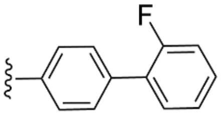	—CH_3	—F	41.72	45.53	81.92	-10.68
Terifluno mide	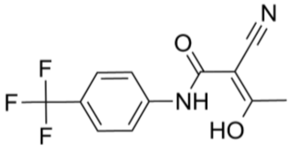			30.78	29.80	55.14	-8.73

Table S3. Percentage of hydrogen bonds (donor-acceptor distance ≤ 3.5 Å).

Compound	H-Acceptor	H-Donor	%	Avg. dist. (Å)
**2a**	LIG359(O1)	ARG136(HH22)	64.18	2.78
	LIG359(O1)	ARG136(HE)	62.19	2.78
	LIG359(C1)	ARG136(HH22)	49.25	3.27
	LIG359(O2)	ARG136(HH22)	38.81	2.89
	LIG359(H4)	PRO364(HD2)	13.93	3.33
	LIG359(C1)	ARG136(HE)	12.94	3.38
	LIG359(H6)	LEU356(HG)	12.44	3.32
	PHE100(HD2)	LIG359(H7)	10.95	3.33
**2e**	GLN47(OE1)	LIG366(H5)	25.87	2.73
	LEU46(HD11)	LIG366(H8)	18.91	3.23
	MET111(HE3)	LIG366(H9)	16.92	3.26
	MET43(O)	LIG366(H5)	14.43	2.85
	LEU46(HD13)	LIG366(H8)	14.43	3.22
	LIG366(H6)	LEU359(HG)	14.43	3.34
	ARG136(HH11)	LIG366(H3)	13.93	3.29
	MET111(HE2)	LIG366(H9)	13.43	3.29
	LIG366(H1)	HIS56(HB2)	12.44	3.29
	MET111(HE1)	LIG366(H9)	11.94	3.19
	LEU46(HD12)	LIG366(H8)	11.94	3.25
	LIG366(H2)	HIS56(HA)	10.95	3.36
	LIG366(H7)	PHE98(HE2)	10.45	3.30
**2d**	GLN47(OE1)	LIG366(H4)	36.32	3.16
	GLY48(O)	LIG366(H14)	22.89	2.71
	LEU46(O)	LIG366(H4)	21.39	3.17
	THR360(O)	LIG366(H14)	17.91	2.88
	PHE98(HZ)	LIG366(H10)	17.41	3.22
	LIG366(H10)	PHE98(HZ)	16.42	3.23
	LIG366(H6)	THR360(HA)	15.92	3.28

	ARG136(HG2)	LIG366(H1)	14.43	3.17
	LIG366(H8)	LEU46(HB1)	13.43	3.34
	LEU46(O)	LIG366(H5)	10.45	3.36
Brequinar	BRQ366(O1)	ARG136(HE)	100.0	2.74
	BRQ366(O2)	ARG136(HH21)	97.01	2.73
	BRQ366(C23)	ARG136(HH21)	92.54	3.22
	BRQ366(C23)	ARG136(HE)	85.57	3.34
	BRQ366(H7)	THR360(HA)	58.21	3.26
	THR360(HA)	BRQ366(C12)	49.25	3.23
	BRQ366(H8)	PRO364(HD2)	25.87	3.29
	PRO364(HD2)	BRQ366(H8)	25.37	3.28
	TYR366(HH)	BRQ366(H6)	23.38	3.24
	BRQ366(H10)	ALA56(HB2)	17.41	3.28
	BRQ366(O2)	ARG136(HE)	16.92	3.22
	BRQ366(O2)	GLN48(HA)	16.92	3.40
	BRQ366(H10)	ALA56(HB3)	15.42	3.27
	BRQ366(H10)	ALA56(HB1)	15.42	3.28
	BRQ366(H4)	PRO53(HB1)	14.93	3.30
	ALA56(HB2)	BRQ366(H10)	13.43	3.23
	ALA56(HB3)	BRQ366(H10)	12.44	3.23
	BRQ366(O1)	ARG136(HH21)	11.94	3.12
	BRQ366(H6)	HIS57(HB2)	11.94	3.20
	ALA56(HB1)	BRQ366(H10)	10.95	3.24
	HIS57(HA)	BRQ366(H6)	10.45	3.29
	TYR356(OH)	BRQ366(H6)	10.45	3.41

Table S4**.** Details for the data collection and statistical results for data processing.

	**2a**	**2d**
Diffraction source	Manacá Beamline	Manacá Beamline
Wavelength (Å)	0.97714	0.977140
Temperature (K)	100	100
Detector	DECTRIS PILATUS3 2M	DECTRIS PILATUS3 2M
Crystal-detector distance (mm)	129.52	129.57
Rotation range per image (°)	0.2 °	0.2 °
Total rotation range (°)	360 °	360 °
Exposure time per image (s)	0.1 s	0.1 s
Space group	$P3_221$	$P3_221$
a, b, c (Å)	90.59 90.59 123.08	90.83 90.83 123.09
α, β, γ (°)	90.00, 90.00, 120.00	90.00, 90.00, 120.00
Resolution range (Å)	48.420-1.336 (1.42-1.336)	48.473-1.505 (1.60-1.505)
Total No. of reflections	2556813 (391612)	1826141 (295140)
No. of unique reflections	255857 (41339)	179767 (28911)
Completeness (%)	99.9 (99.5)	99.8 (98.9)
⟨ I/σ(I)⟩	11.04 (0.71)	8.16 (0.65)
CC half	99.9 (26.8)	99.7 (24.2)

Table S5. Results for structure refinement.

	**2a**	**2d**
Resolution range (Å)	48.420-1.336 (1.371-1.336)	48.473-1.505 (1.544-1.505)
Completeness (%)	99.91 (98.85)	99.82 (97.62)
No. of reflections, working set	125539 (9139)	88433 (6394)
No. of reflections, test set	6608 (481)	4779 (302)
Final $R_{working}$	0.17253	0.17938
Final R_{free}	0.18147	0.18924
No. of non-H atoms		3134
Protein	2865	2767
Ligands	102	100
FMN	31	31
ORO	11	11
Inhibitor	20	26
Other ligands	40	32
Water	312	267
R.m.s. deviations		
Bonds (Å)	0.94	0.52
Angles (°)	1.31	0.87
Average B factors (Å^2)		
Protein	19.8	20.8
Ligands		
FMN	12.3	12.4
ORO	13.7	14.8
Inhibitor	32.6	18.1
Other ligands	43.4	45.5
Water	36.7	34.9
Ramachandran plot		
Most favoured (%)	97	98
Allowed (%)	3	2

Table S6. Retention time and purity of compounds **2a-2f** and **2j-2k**.

Compound	Retention Time (min)	Purity
**2a**	20.48	99.89%
**2b**	21.57	99.04%
**2c**	19.99	99.44%
**2d**	23.88	95.58%
**2e**	21.14	95,11%
**2f**	20.96	99.68%
**2j**	20.11	100%
**2k**	20.14	95.72%

Figure S1. Analysis of A) protein backbone RMSD along simulation time; B) protein backbone RMSD standard deviation and mean values; C) ligand RMSD along simulation time; D) ligand RMSD standard deviation and mean values; all over three replicas of 200 ns production simulations for HsDHODH complexes with compounds 2d, 2e, 2a and brequinar.

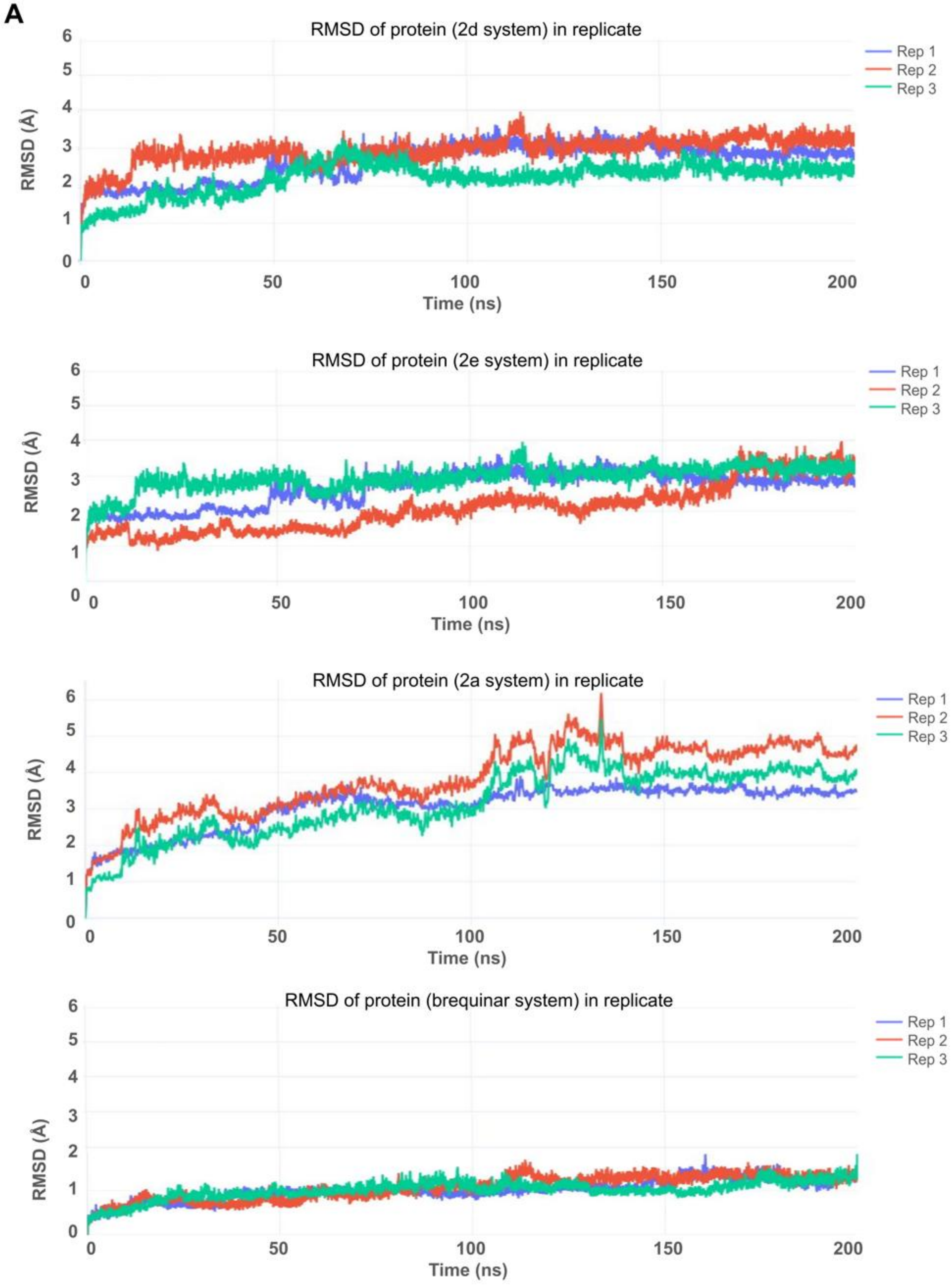

B

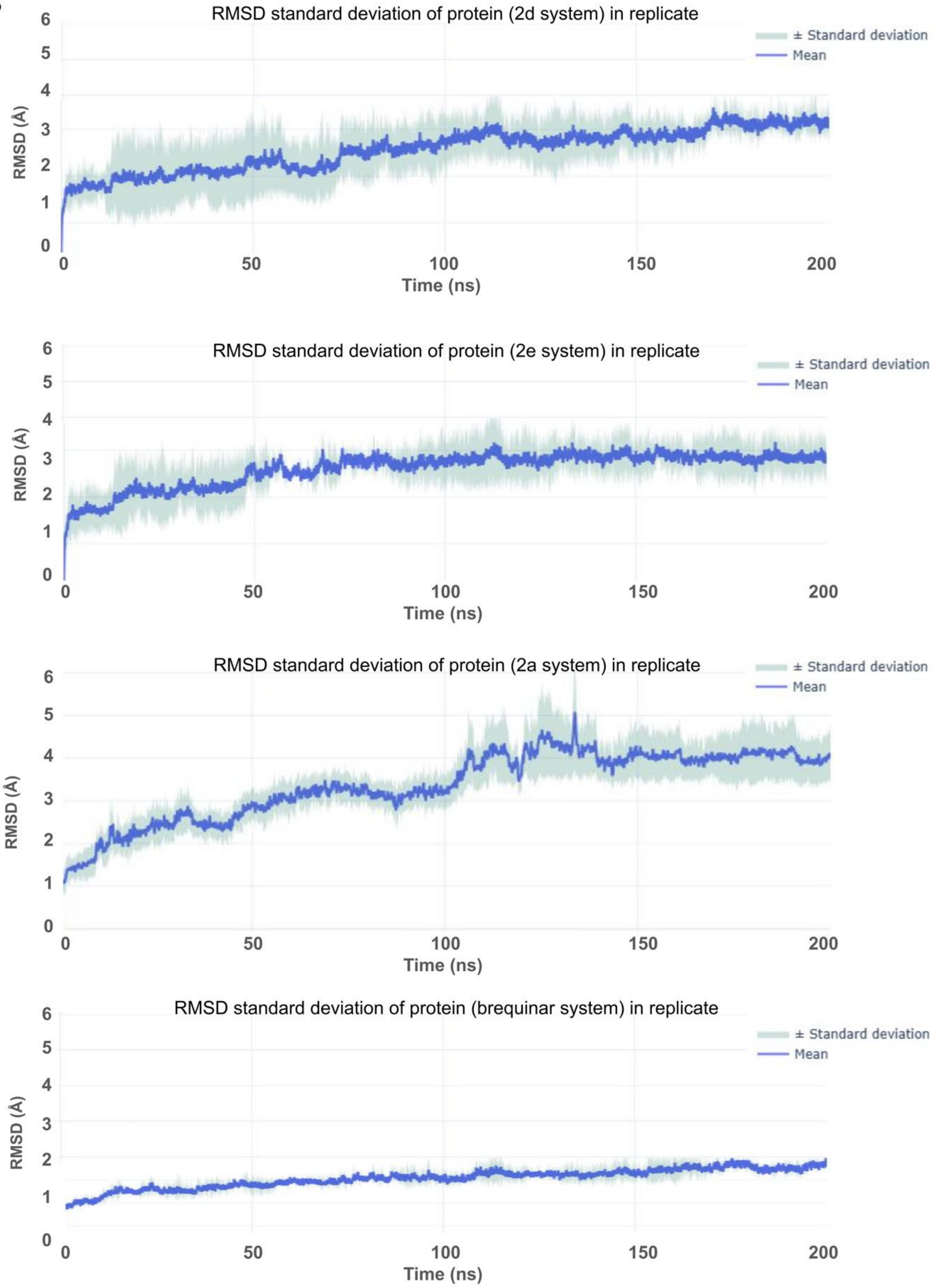

RMSD standard deviation of protein (2d system) in replicate
± Standard deviation
Mean
RMSD (Å)
Time (ns)
0
1
2
3
4
5
6
0
50
100
150
200
RMSD standard deviation of protein (2e system) in replicate
± Standard deviation
Mean
RMSD (Å)
Time (ns)
RMSD standard deviation of protein (2a system) in replicate
± Standard deviation
Mean
RMSD (Å)
Time (ns)
RMSD standard deviation of protein (brequinar system) in replicate
± Standard deviation
Mean
RMSD (Å)
Time (ns)

C

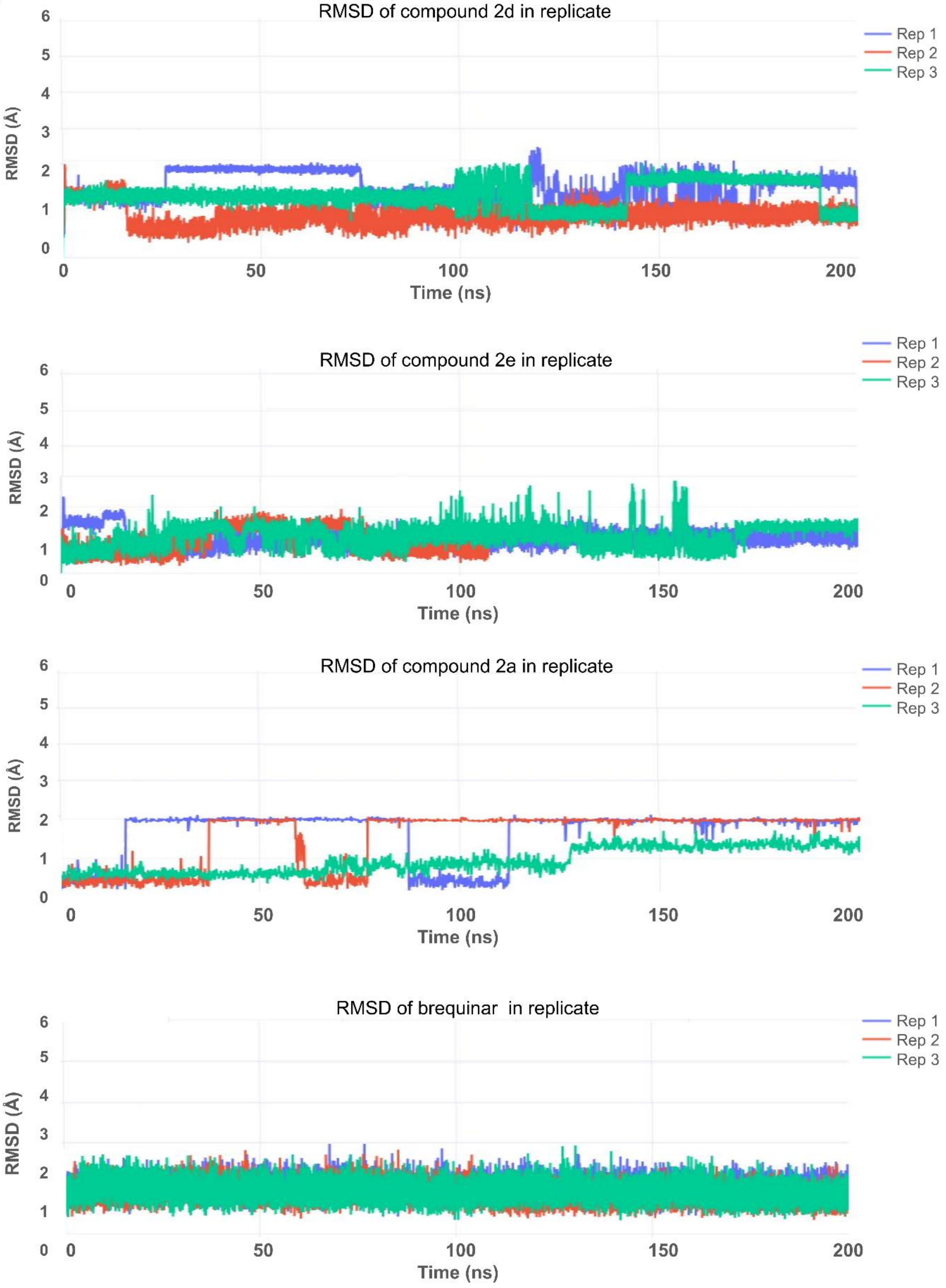
RMSD of compound 2d in replicate
RMSD (Å)
Time (ns)
Rep 1
Rep 2
Rep 3
RMSD of compound 2e in replicate
RMSD (Å)
Time (ns)
Rep 1
Rep 2
Rep 3
RMSD of compound 2a in replicate
RMSD (Å)
Time (ns)
Rep 1
Rep 2
Rep 3
RMSD of brequinar in replicate
RMSD (Å)
Time (ns)
Rep 1
Rep 2
Rep 3

D

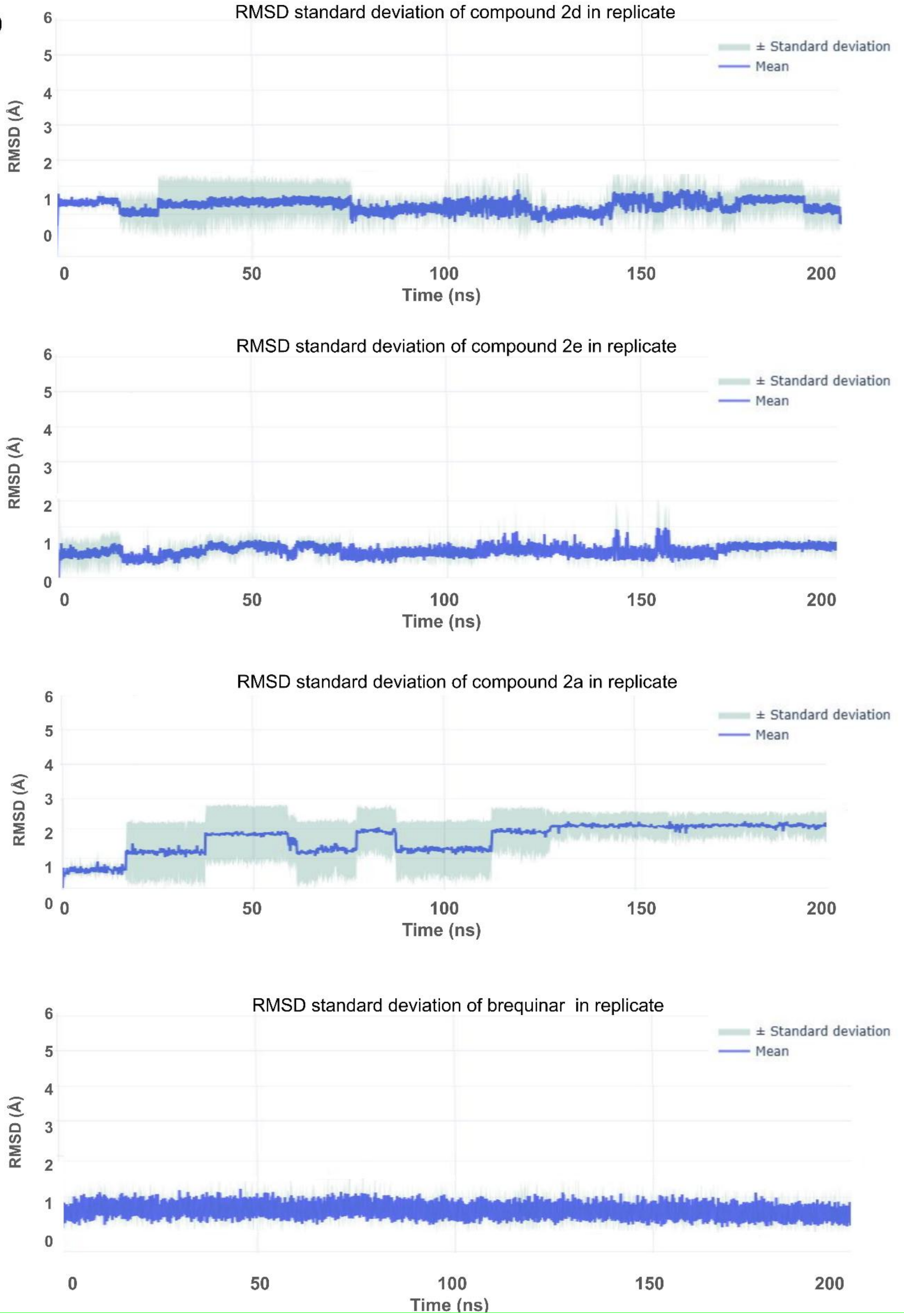
RMSD standard deviation of compound 2d in replicate
± Standard deviation
Mean
RMSD (Å)
Time (ns)
RMSD standard deviation of compound 2e in replicate
± Standard deviation
Mean
RMSD (Å)
Time (ns)
RMSD standard deviation of compound 2a in replicate
± Standard deviation
Mean
RMSD (Å)
Time (ns)
RMSD standard deviation of brequinar in replicate
± Standard deviation
Mean
RMSD (Å)
Time (ns)

Figure S2. Analysis of A) protein RMSF per residues; and B) protein RMSF standard deviation and mean values, all over three replicas of 200 ns production simulations for HsDHODH complexes with compounds 2d, 2e, 2a and brequinar.

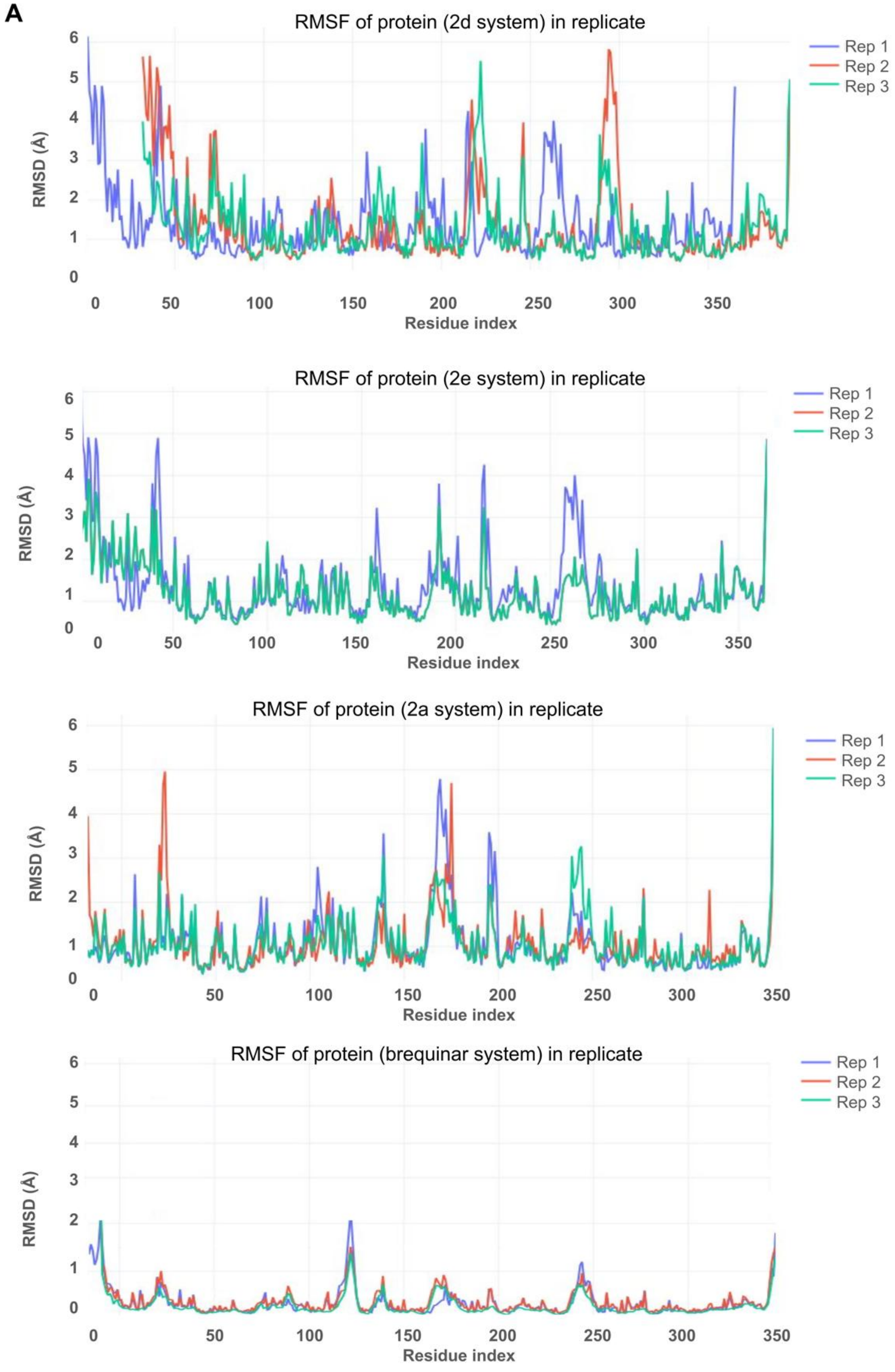

B

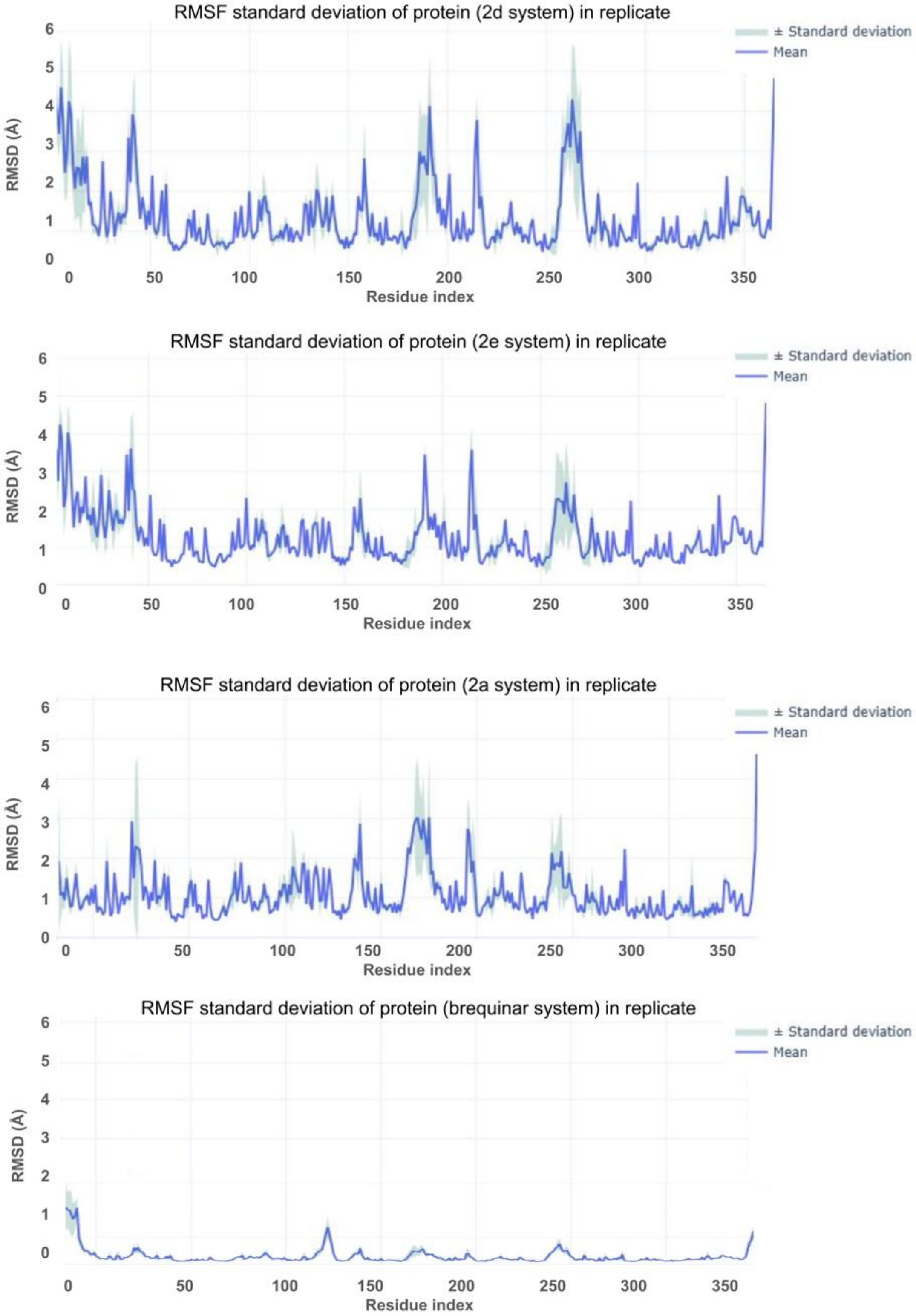
RMSF standard deviation of protein (2d system) in replicate
± Standard deviation
Mean
RMSD (Å)
Residue index
RMSF standard deviation of protein (2e system) in replicate
± Standard deviation
Mean
RMSD (Å)
Residue index
RMSF standard deviation of protein (2a system) in replicate
± Standard deviation
Mean
RMSD (Å)
Residue index
RMSF standard deviation of protein (brequinar system) in replicate
± Standard deviation
Mean
RMSD (Å)
Residue index

Figure S3. Contribution of the residues to the binding energy in kcal/mol. A) Simulation of the *Hs*DHODH complex with compound **2d**; B) Simulation of the *Hs*DHODH complex with compound 2e; C) Simulation of the *Hs*DHODH complex with the brequinar inhibitor; D) Simulation of *Hs*DHODH complex with the compound 2a.

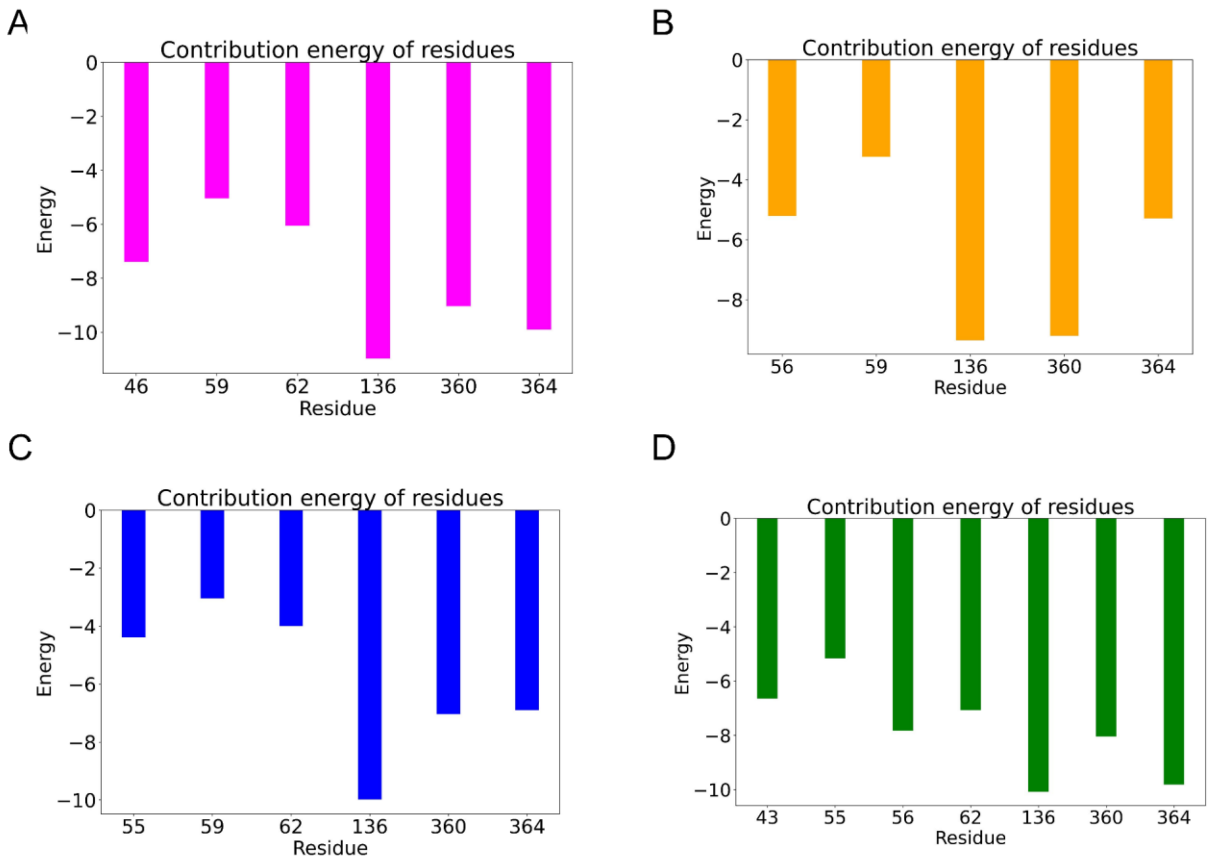

Figure S4. (a) Cartoon representation of the overall fold for the complexes obtained by crystallography and molecular interactions of the inhibitors with *Hs*DHODH, showing the superposition of *Hs*DHODH-2a (salmon) and *Hs*DHODH-2d (blue) complexes, with the C-terminal domain (Met78-Arg396), the linking loop, and the N-terminal domain (Glu35-Leu68). ORO and FMN are shown as sticks and colored orange, and inhibitors are shown as sticks and colored blue to 2d and salmon to 2a. (b) Superposition of 2a and 2d inhibitors in complex with *Hs*DHODH. (c-d) binding site residues involved in interactions, the ligand and the refined maps for the ligand. The residues are shown as sticks and colored wheat for the C-terminal domain residues and pink for N-terminal residues. The ligands and residues involved in hydrophilic interactions are shown as sticks and colored by atoms: oxygen (red), nitrogen (blue), fluor (cyan), and carbon (blue to 2d and salmon to 2a).

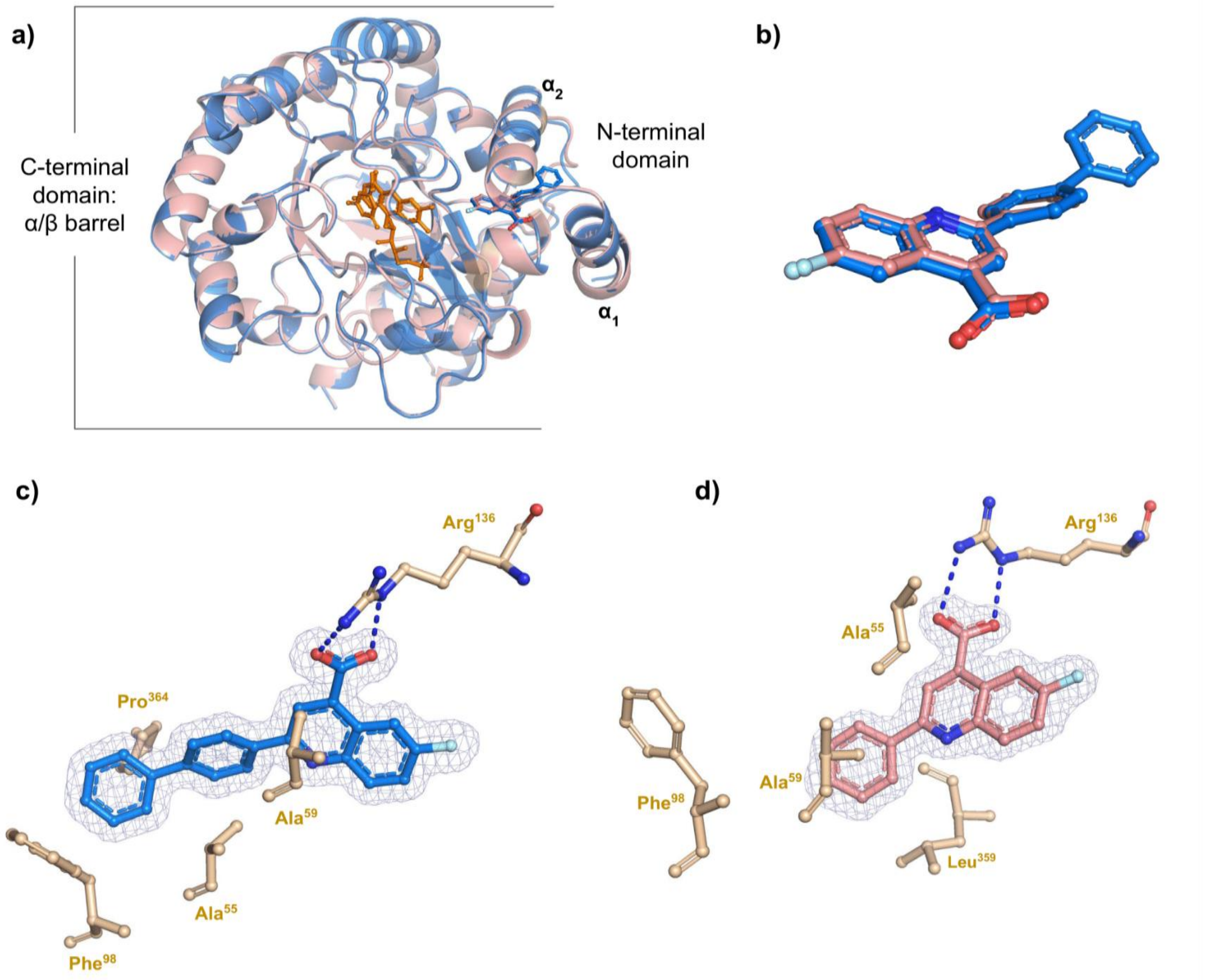

Figure S5. HPLC spectrum of 6-fluoro-2-phenylquinoline-4-carboxylic acid (**2a**)

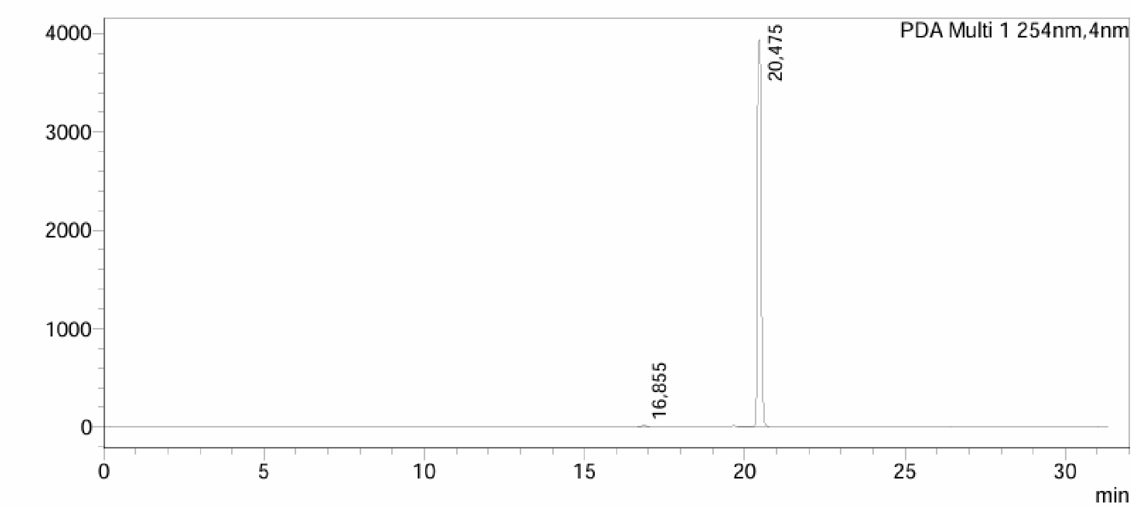

Figure S6. HPLC spectrum of 6-fluoro-2-(p-tolyl)quinoline-4-carboxylic acid (**2b**)

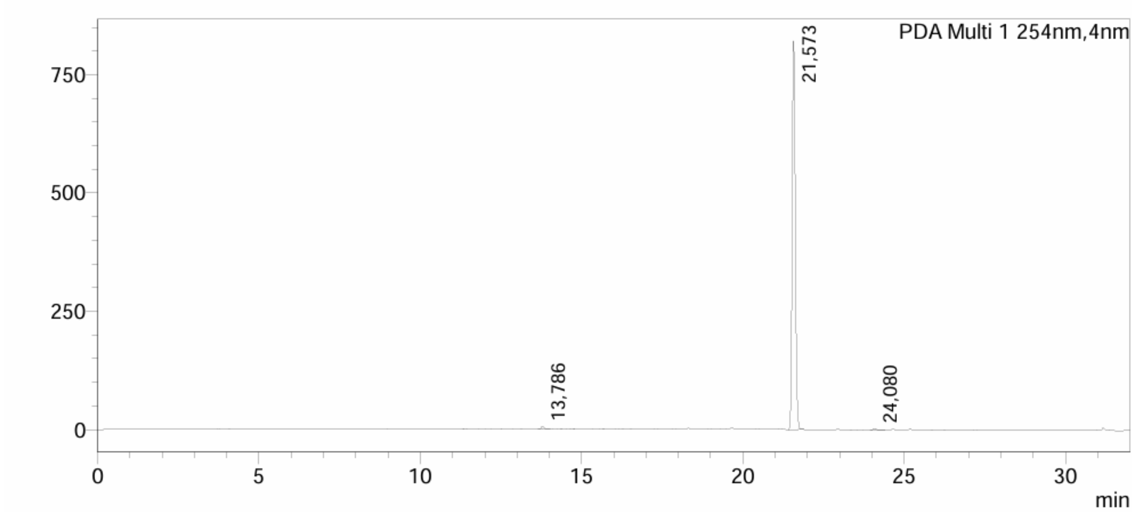

Figure S7. HPLC spectrum of 6-fluoro-2-(o-tolyl)quinoline-4-carboxylic acid (**2c**)

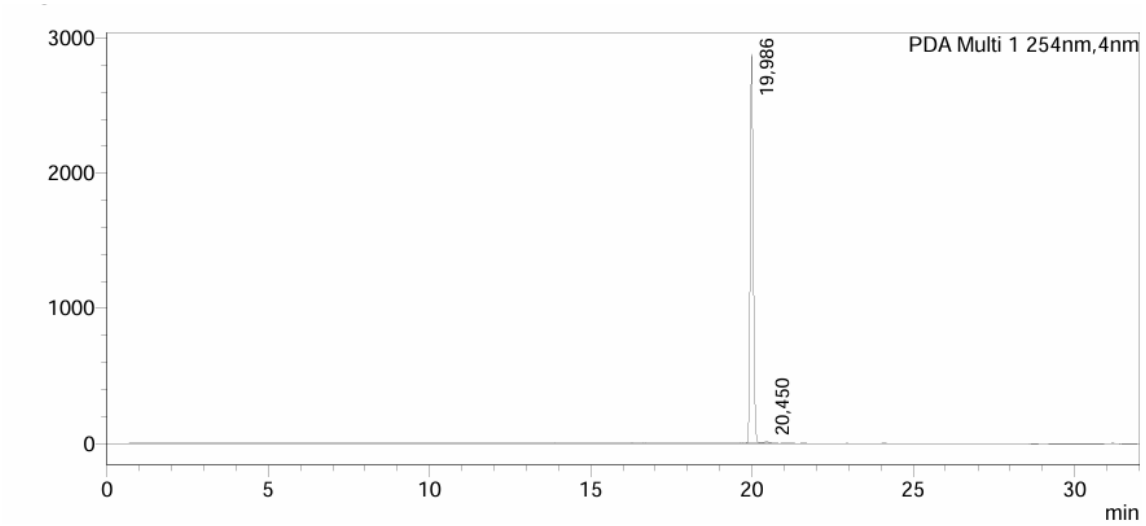

Figure S8. HPLC spectrum of 2-([1,1'-biphenyl]-4-yl)-6-fluoroquinoline-4-carboxylic acid (**2d**)

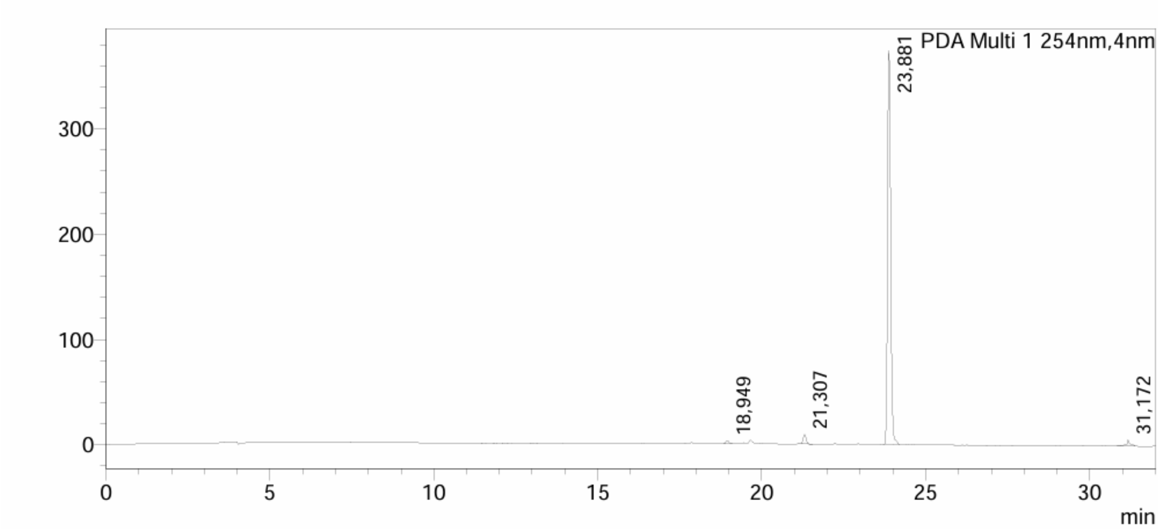

Figure S9. HPLC spectrum of 6-fluoro-2-(naphthalen-2-yl)quinoline-4-carboxylic acid (**2e**)

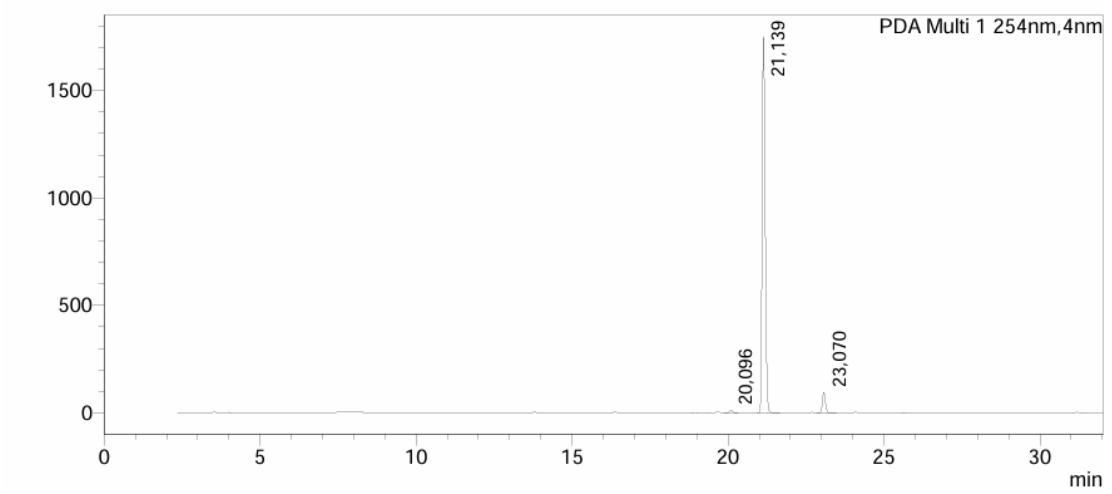

Figure S10. HPLC spectrum of 6-fluoro-2-(4-fluorophenyl)quinoline-4-carboxylic acid (**2f**)

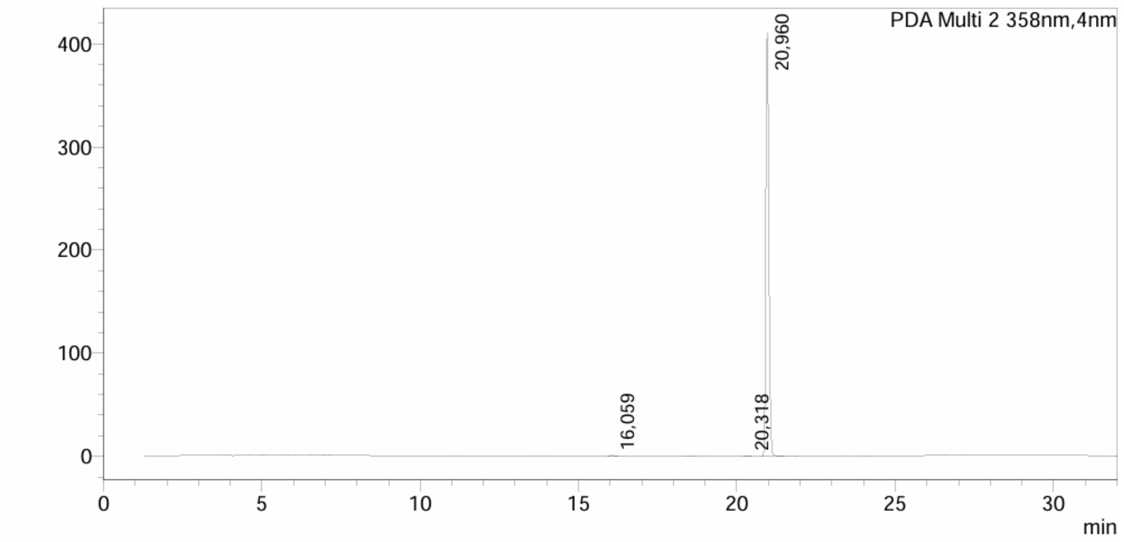

Figure S11. HPLC spectrum of 6-fluoro-2-(4-methoxyphenyl)quinoline-4-carboxylic acid **(2j)**

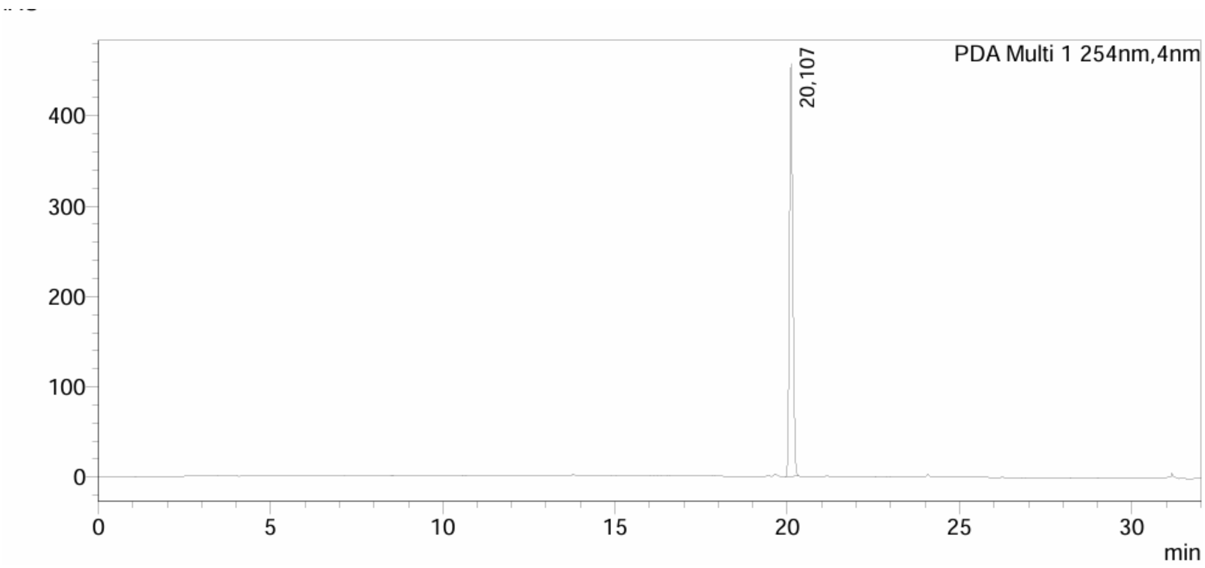

Figure S12. HPLC spectrum of 2-(benzo[d][1,3]dioxol-5-yl)-6-fluoroquinoline-4-carboxylic acid (**2k**)

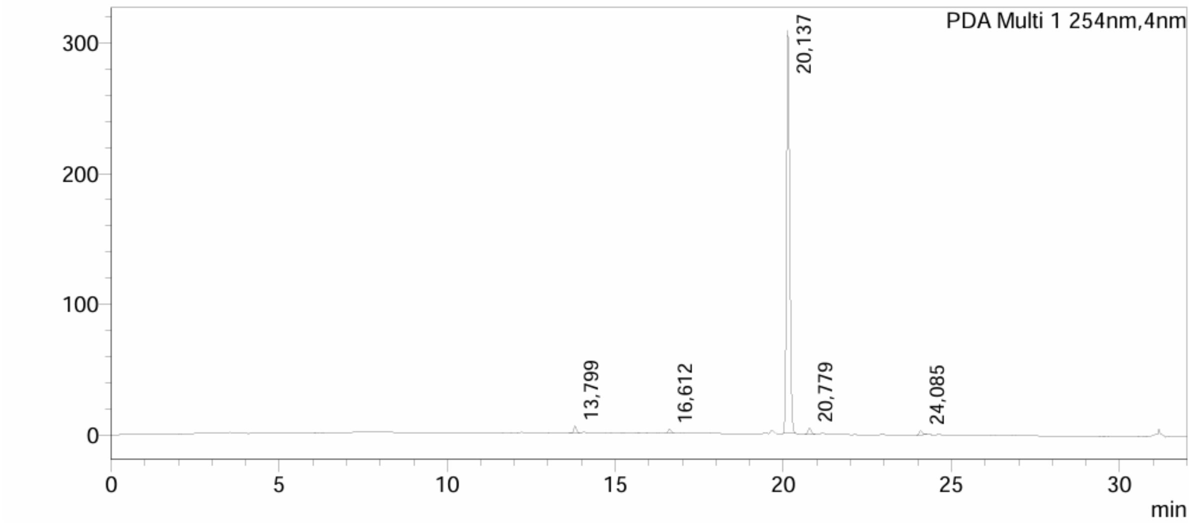

Supplement: Supplementary file 1 [file ao5c12978_si_001.pdf]
